# Supplementary material for: A gender-sensitised weight-loss and healthy living program for men with overweight and obesity in Australian Football League settings (Aussie-FIT): A pilot randomised controlled trial
Source: PLoS Med. 2020 Aug 6;17(8):e1003136. doi: 10.1371/journal.pmed.1003136 (PMC7410214; doi:10.1371/journal.pmed.1003136)
Supplement: S1 Table — (S1A) CONSORT 2010 checklist of information to include when reporting a pilot or feasibility trial. (S1B) The TIDieR Checklist. CONSORT, Consolidated Standards of Reporting Trials; TIDieR, Template for Intervention Description and Replication. (DOCX) [file pmed.1003136.s001.docx]

*S1 Appendix.* Consolidated Standards of Reporting Trials (CONSORT) guidelines extension for randomised pilot and feasibility trials and Template for Intervention Description and Replication (TIDieR) checklists.

**S1A. CONSORT 2010 checklist of information to include when reporting a pilot or feasibility trial***

| **Section/Topic** | **Item No** | **Checklist item** |  |
| --- | --- | --- | --- |
| **Title and abstract** | | | |
|  | 1a | Identification as a pilot or feasibility randomised trial in the title: “A gender-sensitised weight loss and healthy living program for overweight and obese men in Australian Football League settings (Aussie-FIT): A pilot randomised” |  |
|  | 1b | Structured summary of pilot trial design, methods, results, and conclusions (for specific guidance see CONSORT abstract extension for pilot trials): “**Abstract**  **Background:** Recent evidence shows that sport settings can act as a powerful draw to engage men in weight loss. The primary objective of this pilot study was to test feasibility of delivering and evaluating preliminary efficacy of Aussie-FIT, a weight loss program for overweight/obese men delivered in Australian Football League settings, in preparation for a future definitive trial.  **Methods and Findings:** This 6-month pilot trial took place in Perth, Australia. Participants were overweight/obese (BMI > 28 kg/m^2^), middle-aged (35-65 years old) men. The intervention involved 12 weekly 90-minute face-to-face sessions, incorporating physical activity, nutrition, and behaviour change information and practical activities delivered by coaches at two clubs. Data were collected at baseline and immediately post-intervention. For trial feasibility purposes, 6-month follow-ups were completed. Outcomes were differences in weight loss (primary outcome), and recruitment and retention rates, self-reported measures (e.g., psychological well-being), device-measured physical activity, waist size, and blood pressure at 3-months.  Within three days of advertising at each club, 426 men registered interest; 306 (72%) were eligible. Men were selected on first-come first-served basis (*n* = 130; *M* age = 45.78, *SD* = 8.01; *M* BMI = 34.48 kg/m^2^, *SD* = 4.87) and randomised by a blinded researcher. Trial retention was 86% and 63% at 3- and 6-month follow-ups (respectively). At 3 months, mean difference in weight between groups, adjusted for baseline weight and group, was 3.33kg (95% CI 1.89, 4.77) in favour of the intervention group (*p* < .001). The intervention group’s moderate-to-vigorous physical activity was higher than the control group by 8.54 mins/day (95% CI 1.37, 15.71, *p* = .02). No adverse events were reported.  **Conclusions:** Aussie-FIT was feasible to deliver; participants increased physical activity, decreased weight, and reported improvements in other outcomes. Issues with retention were a limitation of this trial. In a future, fully powered randomised controlled trial (RCT), retention could be improved by conducting assessments outside of holiday seasons. |  |
| **Introduction** | | | |
| Background and objectives | 2a | Scientific background and explanation of rationale for future definitive trial, and reasons for randomised pilot trial: “Obesity is a global public health issue and prevalence is increasing; in 2016, over 1.9 billion people worldwide were overweight, and over 650 million were obese [1]. In Australia in 2016, approximately 60% of adults were estimated to be overweight, including 25% classified as obese [2]. Although in Australia overweight and obesity are more prevalent in men than in women (71% versus 56%) [3], men are underrepresented in trials of weight loss, and current community programs have not appealed to them [4, 5]. Prevalence of overweight and obesity in men and low participation in trials may be in part because cultural constructions of masculinity in Western societies often promote overeating, excessive alcohol consumption, and stereotype dieting or healthy eating as a ‘female-only’ activity [6–8]. Weight loss programs are typically marketed to females, and are rarely tailored to attract and meet the needs of men [6, 9].  Professional sports can be used as a powerful draw to attract men to weight loss programs [10, 11]. For example, the Football Fans in Training (FFIT) program was designed to appeal to overweight and obese soccer fans in Scotland and to support them in losing weight, by fostering changes in their dietary and physical activity behaviours [12–14]. The program was delivered over 12 weekly sessions in the context of professional soccer (e.g., premier league stadia, training facilities) and was effective in reducing weight (baseline adjusted mean difference in weight loss between intervention and control groups at 12-month follow up was 4.94 kg). FFIT also improved health behaviours and psychological well-being [14] and these effects were maintained 3.5 years post baseline [15].  FFIT has been successfully adapted for other professional sport settings and for different countries, including rugby in England [16] and New Zealand – RuFIT [17], and hockey in Canada – Hockey FIT [18] and HAT TRICK [19]. The model has also been successfully used to inform healthy lifestyle programs for other outcomes, as in EuroFIT, which focussed on physical activity and sedentary time, delivered in four European countries [20]. To date, this program has not been evaluated in Australia; and programs that address unique masculinity constructs are still an exception in Australia [21].  Building on FFIT, we developed the Australian Fans in Training (Aussie-FIT) program, a culturally sensitised version of FFIT for men in Australia, for delivery in Australian Football League (AFL) settings. The key features of Aussie-FIT have been described elsewhere [22]. Key innovations included the program being further developed in the context of behaviour change theories summarised in a recent theory review [23], and on reviews of successful studies of weight loss maintenance [24–25]. This included the integration of principles from Self-Determination Theory (SDT) as a means to strengthen the quality, quantity, and longevity of motivation to change health behaviours [26]. According to SDT, humans have three psychological needs. These include the need to feel that one is competent (i.e., has the skills, support and resources to meet challenges or strive for goals), autonomous (i.e., that what one is doing reflects personal choice, values and volition), and related to others (i.e., respected, supported, cared for and connected). A plethora of studies have indicated health behaviour change to be more effective and well-being to be enhanced when these needs are satisfied. Need satisfaction is important to foster as it almost unequivocally predicts more autonomous (i.e., self-determined, valued, and relevant) motives [26] which, according to a recent systematic review [27], are strongly related to long-term weight maintenance and physical activity behaviour. Accordingly, principles of SDT were applied throughout the program via integration into coach training and program activities. In line with theoretical understanding [23] and previous studies [25], men need to effectively self-regulate their behaviour, and successfully form healthy habits to maintain long-term behaviour change. Social support and environmental factors play a crucial role in health behaviour change and long-term maintenance [9, 21]. Men need access to plentiful resources to maintain their behaviour long-term, including both physical (access to healthy food, exercise equipment) and psychological (energy levels, positive mood) and social resources to facilitate health behaviour change maintenance [28, 29]. |  |
|  | 2b | Specific objectives or research questions for pilot trial: “The overarching aim of the study was to test the feasibility of delivering and evaluating the 12-week Aussie-FIT program and preliminary efficacy in the context of AFL in Australia to determine the appropriateness of a future randomised controlled trial (RCT). Specifically, the primary objectives were to examine: (1) the feasibility of recruiting the target sample and retaining them at 3- and 6-month follow ups; (2) the appropriateness of the selected measures to examine effects, and to report effects of the intervention at 3-month follow up on body weight, waist circumference, blood pressure; device measured time spent in moderate/vigorous PA and sedentary time; self-reported dietary screener, and a range of secondary psychological outcomes; (3) the feasibility of administering the data collection protocols and questionnaire administration procedures in the AFL club setting at all three time points; and (4); to assess the feasibility of collecting data to develop and undertake preliminary tests of a model to determine cost-effectiveness of the program. Additional feasibility outcomes will be examined in a full process evaluation, reported elsewhere.” |  |
| **Methods** | | | |
| Trial design | 3a | Description of pilot trial design (such as parallel, factorial) including allocation ratio: “We undertook a two-group pilot waitlist RCT from April-December 2018 in the Perth metropolitan area of Western Australia. The waitlist control treatment was chosen to align with FFIT RCT design and to minimise any ethical concerns related to withholding treatment in the control group. Participating groups were stratified by site (two) and measures were collected at baseline, and 3, and 6 months post baseline. The waitlist control group started participation in the program at 3 months; therefore, the 6-month measure represents a ‘post-program’ assessment for the control arm, and a short-term maintenance effect for the intervention arm.” |  |
|  | 3b | Important changes to methods after pilot trial commencement (such as eligibility criteria), with reasons – Not applicable |  |
| Participants | 4a | Eligibility criteria for participants: “The study inclusion criteria were: men aged 35-65 years, with BMI 28kg/m^2^ or higher (eligibility based on baseline measures). Age and BMI cut-offs were based on the FFIT study [32]. The study exclusion criteria were: men who were unable to comprehend English; unable to provide informed consent and/or agree to the randomisation; and those who were already participating in a health promotion or weight loss program.” |  |
|  | 4b | Settings and locations where the data were collected: “Measures were collected at the two AFL club facilities where we also delivered the Aussie-FIT sessions (alternative arrangements were made for men who could not attend measurement sessions at the club (e.g., meetings at university premises).” |  |
|  | 4c | How participants were identified and consented: “Men were recruited through a variety of methods, including features in weekly fan emails from the participating AFL clubs, social media announcements from the clubs and directly from the Aussie-FIT project social media accounts, word of mouth, and via the Aussie-FIT website [33]. We invited potential participants to register their interest and complete an eligibility check by visiting the program website and providing their age, weight, height, contact details, and availability to attend Aussie-FIT sessions.”  “After a brief welcome and summary of what the assessment session involved, men provided informed consent and completed measures at supervised stations” |  |
| Interventions | 5 | The interventions for each group with sufficient details to allow replication, including how and when they were actually administered: “Aligned with the FFIT program, the Aussie-FIT intervention included 12 weekly 90 minute sessions designed to promote physical activity, healthy eating, and weight loss. The program was delivered to groups of approximately 15 men by one coach and included classroom-based activities and physical activity sessions. In the early weeks, a little more than half of the session was dedicated to classroom activities, with less time allotted to the physical activity sessions. Over the course of 12 weeks, the balance shifted to expand the physical activity component to align with the men’s progress in fitness. The delivery style was informal, encouraging positive social interaction, humour, and ‘friendly banter’. The program was gender-sensitised with an emphasis on dietary and physical activity changes that are consistent with masculinised practices (e.g., ordering salad with a steak, getting back to being able to play football with children or grandchildren, discussion of the role of alcohol in weight loss, and fostering group support). The program supported participants to make small sustainable changes to their eating through portion control, reduced consumption of sugary drinks, energy-dense foods and alcohol, and a gradual increase in physical activity by choosing the activity that the men enjoy the most or could most easily incorporate into daily life. To make the program culturally appropriate, program content was adapted to reflect the Australian Guidelines for Healthy Eating [36], and resources from ‘LiveLighter’ (a healthy eating campaign in Australia) were built into the program to illustrate key principles (e.g., tool for ease of reading food labels). The program was designed to teach participants strategies for self-regulation, goal setting, and avoiding compensatory behaviours (e.g., overeating after intense physical activity), and to prevent relapse.  Building on the FFIT program [14], Aussie-FIT added new content to both coach training and intervention content. In particular, new coach training included information on what coaches can say and do to create more need supportive and less controlling environments, and why. This innovation was added as need support is known to promote self-determined motivation for behaviour change [37] and increased focus on this aspect of the intervention could further contribute to behaviour change and maintenance. This content was delivered in coach training with discussion of the basic principles of the theory, interactive activities (e.g., scenarios, role playing), detailed descriptions of these environmental components and specific planning activities in which coaches detailed in writing what they planned to do during each session to be need supportive, and how.  The program also included specific activities in which participants reflected on their personal motives and identified sources of basic psychological need satisfaction. In-session activities were included that encouraged men to recognise their own experiences of need satisfaction and sources of autonomous motivation. Previous studies reported that focus on weight loss maintenance should be incorporated from the beginning of the weight loss program [23–24, 38]; therefore, we placed greater emphasis from the start on relapse prevention and on long-term maintenance of behavioural changes. Building on the content of the original FFIT program, Aussie-FIT participants were supported in how to best form habits [14], and how to form specific action and coping plans (expanding on their initial SMART goals); these plans were re-visited and revised during subsequent Aussie-FIT sessions.  Other innovative aspects of the Aussie-FIT intervention included participants and coaches being invited to join closed Facebook groups which comprised the ~15 men in their Aussie-FIT group and their coach. Automated text messages, written in language to promote feelings of autonomy, competence, and relatedness, were sent each week to encourage session attendance and included a brief description of the topic of the upcoming session (e.g. “*Today we will talk about junk food – can be tough to cut it out altogether, but there are plenty of ways you can make it healthier”*). In session one, participants received an Aussie-FIT booklet with session summaries, space to complete in-session activities and to self-monitor their weight loss progress and goals. Men also received activity monitors (Fitbit Zip), club t-shirts, and reusable ‘LiveLighter’ branded water bottles. Additional session information (e.g., summaries of key points covered in the program) was available online, via a password protected subsection of the program website.” |  |
| Outcomes | 6a | Completely defined prespecified assessments or measurements to address each pilot trial objective specified in 2b, including how and when they were assessed: “**Measures**  The primary outcome in this trial was mean difference in weight between groups at 3 months, adjusted for baseline weight. Secondary outcome measures were feasibility of recruitment and trial retention, device-measured weight, waist and blood pressure, physical activity, self-reported diet and alcohol, well-being, quality of life, motivation for physical activity and other variables relevant to behaviour change, and self-reported sleep.  ***Feasibility of recruitment and trial retention.*** Recruitment rates were recorded as the number of men who registered interest in the trial either via a web-based form or over the phone. The online form included a self-administered screening tool to check men’s age and BMI met the inclusion criteria. We also recorded the number of men who were retained to the randomisation stage and those who attended follow ups at 3 and 6 months. Feasibility-related outcomes are reported descriptively.  ***Device-measured weight, waist and blood pressure.*** Weight was assessed using an electronic scale (Seca 813 Flat Scale, EMSE81, Birmingham, United Kingdom). Waist circumference was measured twice, or three times if two measures differed by more than 5 mm; at each time point we took the average of these measures. Resting blood pressure was measured with a digital blood pressure monitor (Omron HEM-705CP, Milton Keynes, UK) after sitting for at least 5 min. If systolic blood pressure was over 139 mm/Hg and/or diastolic blood pressure was over 89 mm/Hg, two further measures were taken, recorded, and a mean calculated from the second and third measures.  ***Physical activity.*** To provide a device-based measure of physical activity and sedentary behaviour/time; participants were fitted with a hip-worn ActiGraph GTX-9 (ActiGraph, Pensacola, the USA) [41] accelerometer which they were asked to wear continuously (24 h/day) for the following eight days. The GTX-9 was programmed to record raw data at a frequency of 30Hz, which were later reduced to vertical axis movement counts of 60s epoch for the purpose of the current analyses. Participants were instructed to wear the accelerometer on the right hip continuously, except during bathing or aquatic activities. Accelerometer data were downloaded using ActiLife version 6.5.4, and one minute epoch data were processed using a validated algorithm in SAS (version 9.3) [42]. Common cut-points [43, 44] were used to classify each minute as sedentary (<100 counts per minute, cpm), light intensity (100–1951 cpm), moderate intensity (1952–5724 cpm), or vigorous intensity (>5724 cpm). The uncensored step count (i.e., all steps counted) recorded per minute was also used. All participants with ≥4 valid days of data were included in the analyses of physical activity and sedentary behaviour.  ***Self-reported dietary screener and alcohol assessment.*** Nutrition relevant outcomes were assessed using the Dietary Instrument for Nutrition Education-based measures [45] adapted for the Australian population [46]. Briefly, the dietary screener asked participants to report how many times over the past 7 days they ate or drank specific foods. From the responses, we calculated the average change in fatty food score, fruit and vegetable score, sugary food score (all three on a 1-4 scale with higher scores indicative of higher consumption). We used a 7-day recall method to measure alcohol consumption and based on the calendar recall data we calculated total alcohol consumption (reported as total average number of alcohol units consumed in a week; one unit is 10ml of pure alcohol).  ***Well-being, quality of life and motivation-related variables.*** We used the 10-item Rosenberg self–esteem scale (range 1-4), where higher scores indicate higher self-esteem [47], the 10-item Short Form of the Positive and Negative Affect Scale (PANAS) separating the two constructs [48] (range 1-5 for both), where higher scores indicate higher positive or negative affect. The 12-item Interpersonal Behaviours Questionnaire (IBQ) was applied to assess men’s perceptions of the psychological need support in relation to weight loss that they received from family and friends (range 1-7), with a higher score indicating more support [49]. We used items from an adapted measure of the Treatment Self-Regulation Questionnaire of weight loss motivation [50] to assess autonomous and controlled motivation regulating weight loss behaviours (range 1-5), where higher scores indicate higher autonomous motivation [50]. We assessed autonomy, competence [51], and relatedness [52] psychological need satisfaction in relation to weight loss behaviours, and collapsed all needs into one score for the purposes of the analysis (range 1-5), with higher scores indicative of higher need satisfaction. We measured health-related quality of life using the EuroQol five-dimensional five level version (EQ-5D-5L, range 1-5, mean score), with lower scores indicative of higher health-related quality of life [53] and EQ-5 overall score (range 0-100), where high scores indicate better overall health.  ***Variables relevant to behaviour change.*** We also assessed goal facilitation and competing goals in relation to weight loss goals [54], barriers [55] and planning [56] (all scores range 1-5), and habits using the Self-Report Behavioural Automaticity Index (SRBAI) for physical activity and for healthy eating [57] (both scales range 1-7). For all these measures, higher scores are indicative of improvement on the construct assessed.  ***Self-reported sleep***. We also used the Pittsburgh Sleep Quality Index (PSQI), to assess seven sleep components; a score of zero indicates highest possible quality sleep and 21 indicates the worst sleep quality [58].” |  |
|  | 6b | Any changes to pilot trial assessments or measurements after the pilot trial commenced, with reasons: “We intended to recruit coaches via the AFL clubs, however the clubs did not have coaches readily available to fulfil this role. As a result, three coaches were recruited via recommendations from the clubs and three coaches were independently identified by the research team. All Aussie-FIT coaches’ professional backgrounds (which included teaching, coaching, exercise instruction and sport science) meant they were likely to be equipped to create an environment that is seen as central to the success of FFIT, i.e., non-didactic, encouraging, interactive delivery style that incorporates appropriate banter to support vicarious learning and team spirit [74]. However, those coaches who were not directly associated with the club may have been less able to integrate into their coaching style other characteristics seen as contributing to FFIT’s success, such as the ‘behind the scenes’ stories and tacit knowledge of the inner workings of the club into program delivery [74]. To overcome this, within the coach training, the coaches were encouraged to think about how to incorporate the sport and club ‘feel’ within their delivery style. Based on qualitative data, the level of connection between the coach and the club did not seem to contribute to any variability in quality of experience any more than other relevant variables such as the coaches’ personality and motivation, or differences in facilities available to deliver the program. However, the extent to which the personal and situational characteristics that shape program delivery impact participants’ experiences of the program and observed outcomes could be further explored in future research, to help inform the most appropriate implementation model for Australia.” |  |
|  | 6c | If applicable, prespecified criteria used to judge whether, or how, to proceed with future definitive trial: “Our findings support progression to a fully powered RCT to further test the effectiveness of the Aussie-FIT program; a future trial should be powered with data from this pilot rather than the FFIT study, and that will also be powered to detect changes in secondary outcomes. The long-term goal of the Aussie-FIT program is to promote long-term maintenance of behaviour changes that have led to weight loss; therefore, longer-term follow-up studies are required to determine whether specific additions included in this program have an impact on long-term outcomes. For instance, FFIT program participants maintained intervention effects at 3.5 years post baseline [2.90 kg (95% CI 1.78, 4.02 kg), *p* < 0.001] [15]; further exploration is needed to assess if the inclusion of specific SDT techniques and BCTs adds value that corresponds with further weight reduction or weight maintenance at a desired level.” |  |
| Sample size | 7a | Rationale for numbers in the pilot trial: “The Aussie-FIT pilot study was designed to inform the development of a definitive future trial. The study sample size followed guidelines for pilot RCTs [30, 31]” |  |
|  | 7b | When applicable, explanation of any interim analyses and stopping guidelines: Not applicable |  |
| Randomisation: |  |  |  |
| Sequence  generation | 8a | Method used to generate the random allocation sequence: “The randomisation sequence was generated by a researcher not involved in the trial using SPSS with block sizes of four, stratified by site and BMI category (with four BMI categories: <30; 30-34.9; 35-39.9; >40 kg/m^2^), and concealed until conditions were assigned. After baseline measures were taken, participants were allocated to the intervention or wait-list control group according to the randomisation sequence and informed about their group allocation via email and phone call. At follow-up measurements, we asked participants not to disclose their group allocation to research assistants; the latter were trained to avoid engaging in extensive conversations with the men during the weight assessment. The weight assessments were taken in a separate area, for participant privacy and also to help mask assessors from hearing men mention their experiences of the program at follow ups. An independent researcher blinded to the study allocation analysed study data. The study is reported in line with Consolidated Standards of Reporting Trials (CONSORT) guidelines extension for randomised pilot and feasibility trials [34] and Template for Intervention Description and Replication (TIDieR) checklist [35](S1 Appendix).” |  |
|  | 8b | Type of randomisation(s); details of any restriction (such as blocking and block size): “The randomisation sequence was generated by a researcher not involved in the trial using SPSS with block sizes of four, stratified by site and BMI category (with four BMI categories: <30; 30-34.9; 35-39.9; >40 kg/m^2^), and concealed until conditions were assigned. After baseline measures were taken, participants were allocated to the intervention or wait-list control group according to the randomisation sequence and informed about their group allocation via email and phone call. At follow-up measurements, we asked participants not to disclose their group allocation to research assistants; the latter were trained to avoid engaging in extensive conversations with the men during the weight assessment. The weight assessments were taken in a separate area, for participant privacy and also to help mask assessors from hearing men mention their experiences of the program at follow ups. An independent researcher blinded to the study allocation analysed study data. The study is reported in line with Consolidated Standards of Reporting Trials (CONSORT) guidelines extension for randomised pilot and feasibility trials [34] and Template for Intervention Description and Replication (TIDieR) checklist [35](S1 Appendix).” |  |
| Allocation  concealment  mechanism | 9 | Mechanism used to implement the random allocation sequence (such as sequentially numbered containers), describing any steps taken to conceal the sequence until interventions were assigned: “The randomisation sequence was generated by a researcher not involved in the trial using SPSS with block sizes of four, stratified by site and BMI category (with four BMI categories: <30; 30-34.9; 35-39.9; >40 kg/m^2^), and concealed until conditions were assigned. After baseline measures were taken, participants were allocated to the intervention or wait-list control group according to the randomisation sequence and informed about their group allocation via email and phone call. At follow-up measurements, we asked participants not to disclose their group allocation to research assistants; the latter were trained to avoid engaging in extensive conversations with the men during the weight assessment. The weight assessments were taken in a separate area, for participant privacy and also to help mask assessors from hearing men mention their experiences of the program at follow ups. An independent researcher blinded to the study allocation analysed study data. The study is reported in line with Consolidated Standards of Reporting Trials (CONSORT) guidelines extension for randomised pilot and feasibility trials [34] and Template for Intervention Description and Replication (TIDieR) checklist [35](S1 Appendix).” |  |
| Implementation | 10 | Who generated the random allocation sequence, who enrolled participants, and who assigned participants to interventions: “The randomisation sequence was generated by a researcher not involved in the trial using SPSS with block sizes of four, stratified by site and BMI category (with four BMI categories: <30; 30-34.9; 35-39.9; >40 kg/m^2^), and concealed until conditions were assigned. After baseline measures were taken, participants were allocated to the intervention or wait-list control group according to the randomisation sequence and informed about their group allocation via email and phone call. At follow-up measurements, we asked participants not to disclose their group allocation to research assistants; the latter were trained to avoid engaging in extensive conversations with the men during the weight assessment. The weight assessments were taken in a separate area, for participant privacy and also to help mask assessors from hearing men mention their experiences of the program at follow ups. An independent researcher blinded to the study allocation analysed study data. The study is reported in line with Consolidated Standards of Reporting Trials (CONSORT) guidelines extension for randomised pilot and feasibility trials [34] and Template for Intervention Description and Replication (TIDieR) checklist [35](S1 Appendix).” |  |
| Blinding | 11a | If done, who was blinded after assignment to interventions (for example, participants, care providers, those assessing outcomes) and how: “At follow-up measurements, we asked participants not to disclose their group allocation to research assistants; the latter were trained to avoid engaging in extensive conversations with the men during the weight assessment. The weight assessments were taken in a separate area, for participant privacy and also to help mask assessors from hearing men mention their experiences of the program at follow ups. An independent researcher blinded to the study allocation analysed study data.” |  |
|  | 11b | If relevant, description of the similarity of interventions: “Aligned with the FFIT program, the Aussie-FIT intervention included 12 weekly 90 minute sessions designed to promote physical activity, healthy eating, and weight loss. The program was delivered to groups of approximately 15 men by one coach and included classroom-based activities and physical activity sessions. In the early weeks, a little more than half of the session was dedicated to classroom activities, with less time allotted to the physical activity sessions. Over the course of 12 weeks, the balance shifted to expand the physical activity component to align with the men’s progress in fitness. The delivery style was informal, encouraging positive social interaction, humour, and ‘friendly banter’. The program was gender-sensitised with an emphasis on dietary and physical activity changes that are consistent with masculinised practices (e.g., ordering salad with a steak, getting back to being able to play football with children or grandchildren, discussion of the role of alcohol in weight loss, and fostering group support). The program supported participants to make small sustainable changes to their eating through portion control, reduced consumption of sugary drinks, energy-dense foods and alcohol, and a gradual increase in physical activity by choosing the activity that the men enjoy the most or could most easily incorporate into daily life. To make the program culturally appropriate, program content was adapted to reflect the Australian Guidelines for Healthy Eating [36], and resources from ‘LiveLighter’ (a healthy eating campaign in Australia) were built into the program to illustrate key principles (e.g., tool for ease of reading food labels). The program was designed to teach participants strategies for self-regulation, goal setting, and avoiding compensatory behaviours (e.g., overeating after intense physical activity), and to prevent relapse.  Building on the FFIT program [14], Aussie-FIT added new content to both coach training and intervention content. In particular, new coach training included information on what coaches can say and do to create more need supportive and less controlling environments, and why. This innovation was added as need support is known to promote self-determined motivation for behaviour change [37] and increased focus on this aspect of the intervention could further contribute to behaviour change and maintenance. This content was delivered in coach training with discussion of the basic principles of the theory, interactive activities (e.g., scenarios, role playing), detailed descriptions of these environmental components and specific planning activities in which coaches detailed in writing what they planned to do during each session to be need supportive, and how.  The program also included specific activities in which participants reflected on their personal motives and identified sources of basic psychological need satisfaction. In-session activities were included that encouraged men to recognise their own experiences of need satisfaction and sources of autonomous motivation. Previous studies reported that focus on weight loss maintenance should be incorporated from the beginning of the weight loss program [23–24, 38]; therefore, we placed greater emphasis from the start on relapse prevention and on long-term maintenance of behavioural changes. Building on the content of the original FFIT program, Aussie-FIT participants were supported in how to best form habits [14], and how to form specific action and coping plans (expanding on their initial SMART goals); these plans were re-visited and revised during subsequent Aussie-FIT sessions.  Other innovative aspects of the Aussie-FIT intervention included participants and coaches being invited to join closed Facebook groups which comprised the ~15 men in their Aussie-FIT group and their coach. Automated text messages, written in language to promote feelings of autonomy, competence, and relatedness, were sent each week to encourage session attendance and included a brief description of the topic of the upcoming session (e.g. “*Today we will talk about junk food – can be tough to cut it out altogether, but there are plenty of ways you can make it healthier”*). In session one, participants received an Aussie-FIT booklet with session summaries, space to complete in-session activities and to self-monitor their weight loss progress and goals. Men also received activity monitors (Fitbit Zip), club t-shirts, and reusable ‘LiveLighter’ branded water bottles. Additional session information (e.g., summaries of key points covered in the program) was available online, via a password protected subsection of the program website.” |  |
| Statistical methods | 12 | Methods used to address each pilot trial objective whether qualitative or quantitative: “Statistical analyses included descriptive statistics and percentages for each group. We used a one-way random effects analysis of covariance in Mplus 8.2 [59] to estimate the treatment effect between groups [60, 61], adjusting for baseline values of the dependent variables and clustering effects (teams of 15 men). We did not stratify by club as we only had two clubs participating, both based in Perth, Western Australia. We conducted an all-cases analysis that involved all participants who were randomised to the intervention or control group and provided at least baseline data; missing data were handled using full information maximum likelihood estimation [62]. We compared the baseline characteristics of the participants who dropped out with the characteristics of the participants who completed the full study to check for differences between the two groups. Secondary analyses focused on maintenance effects for the intervention group and treatment effects for the control group; these analyses were conducted on each group separately. In separate, within-group analyses, we modelled the linear effect of time (3 to 6 months) as a predictor of dependent variables, adjusted for baseline values and the clustering effect of group. In so doing, the effect can be interpreted as the average amount of change on the dependent variable between 3 and 6 months.  Economic analysis included developing and piloting an economic model to test procedures and measures that could be used to estimate cost-effectiveness of the program in a future full-scale trial. Direct costs associated with the program included program set-up, promotion, and delivery material costs. As a gauge of preliminary cost effectiveness, the cost per 5% weight reduction and cost per Quality Adjusted Life Years (QALY) were assessed, using the EQ5D-5L data. The values of these data were converted into utility weights on the basis of the preference weights of a pilot sample from the Australian general population [63].” |  |
| **Results** | | | |
| Participant flow (a diagram is strongly recommended) | 13a | For each group, the numbers of participants who were approached and/or assessed for eligibility, randomly assigned, received intended treatment, and were assessed for each objective:  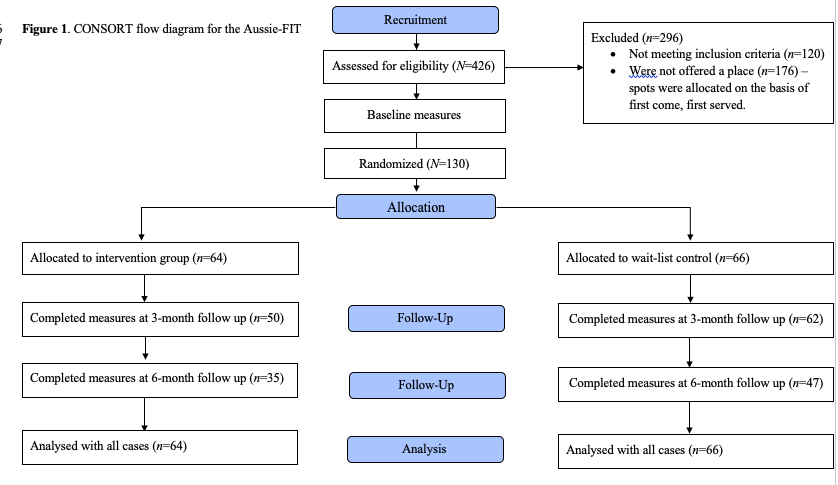 |  |
|  | 13b | For each group, losses and exclusions after randomisation, together with reasons:  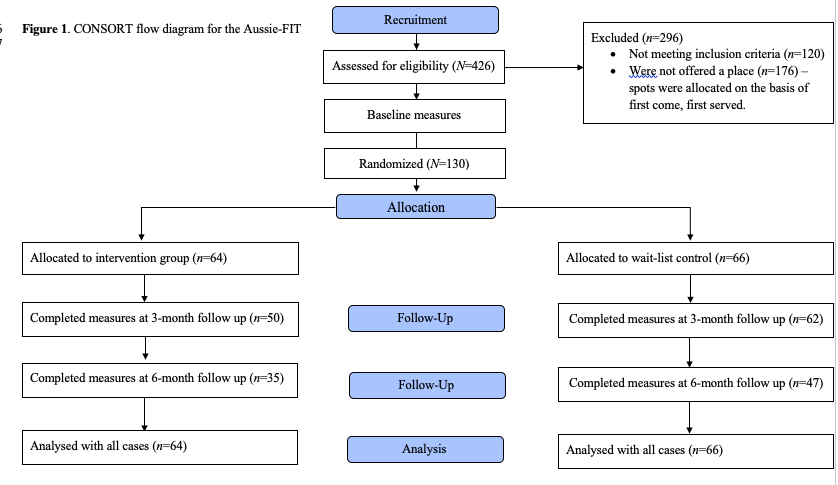 |  |
| Recruitment | 14a | Dates defining the periods of recruitment and follow-up: |  |
|  | 14b | Why the pilot trial ended or was stopped: Not applicable |  |
| Baseline data | 15 | A table showing baseline demographic and clinical characteristics for each group: *Table 1:* Baseline characteristics of participants allocated to the intervention arm or waitlist control arm   \|  \| Aussie-FIT intervention group (n=64) \| Aussie-FIT waitlist control group (n=66) \| Total (N=130) \| \| --- \| --- \| --- \| --- \| \| Age (average years, SD) \| 44.28 (7.68) \| 47.28 (8.04) \| 45.80 (7.98) \| \| Ethnic origin (%)  Caucasian  Mixed  Other \| 61 (95.31)  2 (3.05)  1 (1.52) \| 63 (95.45)  2 (3.15)  1 (1.57) \| 124 (95.4)  4 (3.1)  2 (1.5) \| \| Employment status (%)  In paid employment/self-employed  Retired  Other \| 60 (93.75)  2 (3.05)  2 (3.05) \| 61 (92.42)  0 (0)  5 (7.57) \| 121 (93.07)  2 2 (1.5)  7 (5.38) \| \| Average number of hours worked per week (SD) \| 42.01 (11.74) \| 40.16 (15.12) \| 41.07 (13.54) \| \| Estimated number of days taken off due to illness in the last 6 months (SD) \| 1.32 (1.88) \| 2.46 (4.15) \| 1.90 (3.28) \| \| Number of children (SD) \| 2.08 (1.65) \| 2.03 (1.22) \| 2.05 (1.44) \| \| Years of full-time education (SD) \| 15.09 (3.12) \| 13.00 (2.81) \| 14.03 (3.14) \| \| Housing tenure (%)  Mortgage or loan  Rent  Own  Shared ownership  Live rent free  Other \| 46 (71.90)  9 (14.10)  6 (9.40)  2 (3.10)  0 (0)  1 (1.60) \| 49 (74.24)  6 (9.10)  9 (13.60)  0 (0)  1 (1.57)  1 (1.57) \| 95 (73.07)  15 (11.53)  15 (11.53)  2 (1.5)  1 (0.76)  2 (1.5) \| \| Marital status (%)  Married and living together  Living together but not married  Single  Separated  Divorced  Other \| 49 (76.56)  4 (6.25)  3 (4.68)  6 (9.37)  2 (3.05)  0 (0) \| 50 (75.75)  6 (9.09)  5 (7.57)  0 (0)  3 (4.54)  2 (3.03) \| 99 (76.15)  10 (7.69)  8 (6.15)  6 (4.61)  5 (3.84)  2 (1.5) \| |  |
| Numbers analysed | 16 | For each objective, number of participants (denominator) included in each analysis. If relevant, these numbers should be by randomised group:  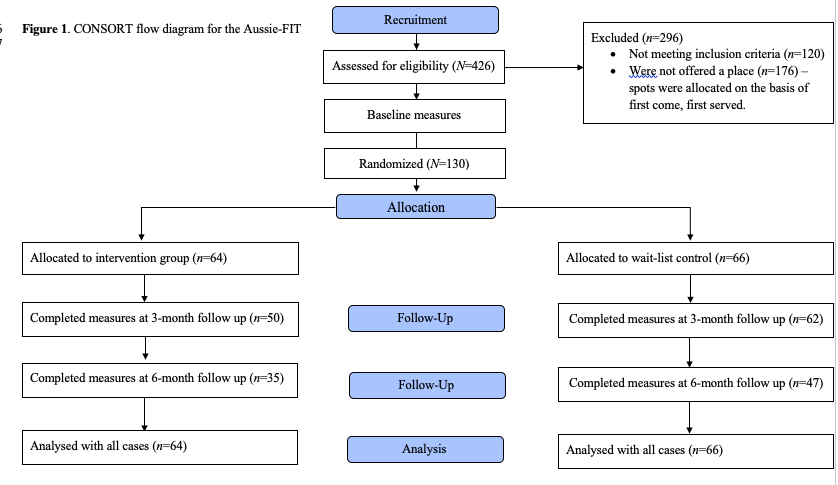 |  |
| Outcomes and estimation | 17 | For each objective, results including expressions of uncertainty (such as 95% confidence interval) for any estimates. If relevant, these results should be by randomised group:  *Table 2:* Group differences in 3- month changes in primary and secondary outcome (per protocol and all cases analysis).   \|  \| **Aussie-FIT intervention group** (baseline n=64)* \| **Aussie-FIT waitlist control group** (baseline n=66) \| **Per protocol (n=112)** \| **All cases analysis (N=130)** \| \| --- \| --- \| --- \| --- \| --- \| \|  \| Group mean \| Group mean \| \| Weight (kg)**  3 months  6 months \| 110.15 (16.37)  107.81 (16.58)  107.21 (18.70) \| 112.64 (19.91)  111.21 (20.60)  108.22 (20.93) \| -3.92 (-6.10, -1.73), p <.001 \| -3.33 (-4.77, -1.89), p <.001 \| \| Weight (% of total body weight lost since baseline)  3 months (from T0 to T1)  6 months (from T0 to T2) \| 3.41 (4.39)  4.51 (6.26) \| 0.53 (3.19)  3.67 (4.58) \| -3.47 (-5.43, -.1.51), p = .001 \| -2.88 (-4.28, -1.48), p <.001 \| \| Waist circumference (cm)  3 months  6 months \| 115.23 (11.79)  113.42 (15.87)  108.73 (13.68) \| 117.24 (15.42)  116.08 (15.51)  110.84 (15.46) \| -2.08 (-6.60, 1.91), p = .32 \| -2.66 (-5.53, .21), p = .07 \| \| BMI (kg/m^2^)  3 months  6 months \| 34.66 (4.63)  33.81 (5.03)  33.71 (5.46) \| 35.32 (6.53)  35.02 (6.99)  34.33 (6.88) \| -1.36 (-2.06, -.66), p < .001 \| -1.48 (-1.73, -.57), p <.001 \| \| Blood pressure (mm/Hg)  Systolic  3 months  6 months  Diastolic  3 months  6 months \| 139.80 (18.81)  136.58 (14.73)  138.29 (14.75)  87.95 (10.31)  87.22 (10.17)  88.69 (7.82) \| 135.95 (14.88)  136.56 (13.13)  136.62 (14.97)  86.67 (9.46)  87.77 (8.64)  88.13 (9.37) \| -4.42 (-9.11, .26), p = .06  -.72 (-4.12, 2.67), p = .68 \| -2.16 (-5.72, 1.41), p = .24  -1.09 (-4.51, 2.33), p = .53 \| \| **Physical activity measured with ActiGraph** \| \| \| \| \| \| Sedentary time (min/day)  3 months  6 months \| 590.74 (94.55)  650.84 (192.32)  536.32 (96.13) \| 571.9 (101.76)  644.22 (171.81)  561.96 (76.19) \| -1.52 (-38.25, 35.20), p = .94 \| -.95 (-35.18, 33.27), p = .96 \| \| LPA time (min/day)  3 months  6 months \| 261.80 (70.21)  264.72 (67.83)  277.37 (79.24) \| 268.2 (71.7)  276.47 (73.01)  298.59 (75.82) \| 2.40 (-29.71, 34.50), p = .88 \| .89 (-21.54, 23.31), p = .94 \| \| MVPA time (min/day)  3 months  6 months \| 35.61 (19.57)  45.14 (22.50)  39.90 (22.50) \| 38.38 (21.5)  37.63 (20.45)  51.00 (31.77) \| 7.86 (-1.25, 16.97), p = .09 \| 8.54 (1.37, 15.71), p = .02 \| \| Steps (uncensored)  3 months  6 months \| 12872 (3770)  13717 (4322)  13762 (4395) \| 13097 (3754)  13421 (4047)  15736 (4620) \| 849.03 (-972.70, 2670.75), p = .36 \| 445.68 (-660.45, 1551.80), p = .43 \| \| Steps (censored)  3 months  6 months \| 10787 (3359)  11654 (4018)  11718 (4049) \| 11053 (3557)  11356 (3828)  13439 (4407) \| 777.35 (-561.04, 2115.75), p = .26 \| 550.37 (-552.24, 1652.97), p = .33 \| \| **Self-reported outcome measures** \| \| \| \| \| \| DINE–based measures (range 1-4):  Fatty food score  3 months  6 months  Fruit and vegetable  3 months  6 months  Sugary food score  3 months  6 months  Total alcohol consumption (units per week)  3 months  6 months \| 1.91 (0.34)  1.70 (0.29)  1.74 (0.29)  1.87 (0.37)  2.24 (0.56)  2.20 (0.47)  1.39 (0.36)  1.10 (0.16)  1.16 (0.29)  8.70 (11.34)  11.50 (8.54)  7.04 (8.95) \| 1.91 (0.31)  1.89 (0.33)  1.61 (0.29)  1.85 (0.53)  2.03 (0.56)  2.28 (0.64)  1.44 (0.47)  1.51 (0.56)  1.16 (0.39)  8.44 (10.88)  8.54 (6.09)  5.81 (5.43) \| -.19 (-.32, -.06), p < .001  .18 (-.06, .42), p = .15  -.39 (-.61, -.18), p < .001  .42 (-.82, 1.66), p = .50 \| -.20 (-.28, -.12), p < .001  .20 (.01, .39), p = .036  -.39 (-.66, -.12), p = .005  1.06 (-.99, 3.11), p = .31 \| \| Self–esteem Rosenberg scale (range 1-4)  3 months  6 months \| 3.02 (0.51)  3.23 (0.51)  3.21 (0.54) \| 2.98 (0.37)  3.03 (0.44)  3.25 (0.42) \| .13 (.02, .24), p = .02 \| .15 (.05, .25), p = .003 \| \| Positive affect (PANAS, range 1-5)  3 months  6 months \| 2.92 (0.70)  3.56 (0.64)  3.35 (0.58) \| 2.89 (0.66)  2.97 (0.66)  3.59 (0.63) \| .58 (.37, .79), p < .001 \| .58 (.35, .80), p < .001 \| \| Negative affect (PANAS, range 1-5)  3 months  6 months \| 1.71 (0.60)  1.53 (0.51)  1.59 (0.54) \| 1.75 (0.56)  1.69 (0.58)  1.48 (0.59) \| -.11 (-.29, .07), p = .25 \| -.14 (-.30, .02), p = .09 \| \| Need support (IBQ scale, range 1-7)  3 months  6 months \| 5.07 (1.18)  5.35 (1.27)  5.33 (1.09) \| 5.33 (1.07)  5.43 (1.13)  5.64 (1.21) \| .18 (-.69, 1.06), p = .68 \| .15 (-.43, .73), p = .61 \| \| Basic need satisfaction in relation to weight loss behaviours (range 1-5)  3 months  6 months \| 3.23 (0.59)  3.92 (0.61)  3.73 (0.75) \| 3.40 (0.65)  3.40 (0.72)  3.97 (0.66) \| .72 (.50, .94), p < .001 \| .60 (.35, .84), p < .001 \| \| Weight loss motivation (range 1-5)  Autonomous  3 months  6 months  Controlled  3 months  6 months \| 4.32 (0.53)  6.32 (0.49)  6.33 (0.67)  2.79 (0.65)  4.26 (1.22)  3.97 (1.07) \| 4.22 (0.55)  6.23 (0.55)  6.50 (0.45)  2.91 (0.58)  4.28 (0.97)  4.10 (1.21) \| .14 (-.05, .32), p = .14  .04 (-.46, .54), p = .88 \| .07 (-.12, .24), p = .48  .11 (-1.22, 1.44), p = .87 \| \| Health-related quality of life (EQ-5, range 1-5)  3 months  6 months \| 1.29 (0.42)  1.27 (0.33)  1.29 (0.34) \| 1.40 (0.39)  1.34 (0.38)  1.31 (0.41) \| .02 (-.14, .18), p = .78 \| -.01 (-.09, .07), p = .81 \| \| Total for health today (range 0-100)  3 months  6 months \| 59.28 (18.63)  74.15 (15.10)  75.37 (13.28) \| 54.92 (16.65)  57.66 (15.82)  69.50 (14.62) \| 16.10 (13.22, 18.97), p < .001 \| 14.57 (9.77, 19.34), p < .001 \| \| Goal facilitation (range 1-5)  3 months  6 months  Competing goals (range 1-5)  3 months  6 months \| 2.64 (0.76)  3.17(0.91)  3.19 (0.75)  3.80 (0.65)  3.38 (0.68)  3.39 (0.75) \| 2.80 (0.75)  2.81 (0.85)  3.41 0.79)  3.67 (0.72)  3.52 (0.71)  3.04 (0.92) \| .54 (-.09, 1.16), p = .09  -.20 (-.48, .07), p = .15 \| .47 (.27, .68), p < .001  -.16 (-.45, .13), p = .27 \| \| Habits for PA (range 1-7)  3 months  6 months  Habits for eating (range 1-7)  3 months  6 months \| 3.18 (1.14)  3.96 (1.31)  4.01 (1.30)  3.02 (1.22)  4.01 (1.30)  3.78 1.35) \| 3.44 (1.27)  3.30 (1.30)  4.18 (1.40)  3.01 (1.07)  2.99 (1.24)  4.05 (1.21) \| 1.07 (.70, 1.45), p < .001  1.17 (.66, 1.57), p < .001 \| .89 (.73, 1.05), p < .001  1.08 (.74, 1.42), p < .001 \| \| Barriers (range 1-5)  3 months  6 months \| 3.16 (0.90)  3.16 (0.78)  3.09 (0.81) \| 3.28 (0.69)  3.14 (0.70)  3.36 (0.83) \| .21 (-.07, .48), p = .14 \| .07 (-.08, .21), p = .38 \| \| Planning (range 1-5)  3 months  6 months \| 3.00 (0.83)  3.70 (0.68)  3.40 (0.92) \| 3.37 (0.86)  3.39 (0.71)  3.82 (0.82) \| .52 (.26, .78), p < .001 \| .39 (.20, .58), p < .001 \| \| Sleep quality (PSQI, range 0-21, lower score indicates a better outcome)  3 months  6 months \| 5.76 (3.05)  4.11 (2.30)  4.77 (3.30) \| 6.59 (3.59)  5.66 (3.02)  4.14 (3.03) \| -1.29 (-2.03, -.55), p = .001 \| -1.18 (-2.16, -.20), p = .02 \|   *Notes.* * n=50 for 3 month measure for intervention group and n=62 for 3 month measure control group; n=35 for 6 month measure for intervention group and n=47 for control group at T2. ** Top line is a baseline measure. Group means are completers means. Censored step counts (n/day) are any steps taken during minutes where the ActiGraph counts per minute were > 100; variance for step count data was larger than the maximum permitted in Mplus (1,000,000), so DV and DVCOV were rescaled by 10 (i.e., linear transformation) for censored and uncensored steps. BMI=body-mass index; LPA – Leisure Time Physical Activity, MVPA – Moderate-and-Vigorous Physical Activity; DINE=dietary instrument for nutritional education; PANAS=positive and negative affect scale. |  |
| Ancillary analyses | 18 | Results of any other analyses performed that could be used to inform the future definitive trial: “The cost effectiveness data were feasible to collect and analyse. The total direct costs associated with the Aussie-FIT program/intervention (i.e., program set-up, promotion, and delivery material costs) amounted to AUD$35,215.38 (i.e. AUD$270.88 per participant), compared to no costs incurred for the comparison group. Thus, the incremental cost per individual was AUD$270.88. The preliminary cost-effectiveness of the Aussie-FIT programme was assessed using two outcome variables: (1) the number of men achieving a 5% weight reduction at 3 months and (2) Quality Adjusted Life Years (QALYs). Thus, we calculated a preliminary indication of the incremental cost-effectiveness (ICER) per each additional man achieving a 5% weight loss at 3 months and the incremental cost-effectiveness per QALY.  At 3 months, 19 out of 64 men (~29.69%) in the intervention group had achieved a 5% weight loss while only 6 out of 66 men (~9.09%) in the comparison group had achieved a 5% weight loss. The incremental effect of the programme concerning a 5% weight loss at 3 months is estimated to be 0.206 (i.e. 0.297-0.091). Thus, ICER per each additional man achieving a 5% weight loss at 3 months was estimated to be AUD$1315.21 (i.e. $\left( \frac{\$270.888}{0.206} \right)$). The EQ-5D-5L values were converted into utility weights on the basis of the preference weights of a pilot sample from the Australian general population, to compute QALYs. At 3 months, the average QALYs for the intervention group were 0.216 (SD=0.041), while for the comparison group it was 0.209 (SD=0.037). The incremental QALY gains at 3 months were estimated to be 0.007 (i.e., 0.216-0.209). Thus, the cost effectiveness of the Aussie-FIT was estimated to be AUD$39,756.42 (i.e. $\left( \frac{\$270.888}{0.007} \right)$) per an additional QALY gained after 3 months.” |  |
| Harms | 19 | All important harms or unintended effects in each group (for specific guidance see CONSORT for harms): Not applicable |  |
|  | 19a | If relevant, other important unintended consequences: Not applicable |  |
| **Discussion** | | | |
| Limitations | 20 | Pilot trial limitations, addressing sources of potential bias and remaining uncertainty about feasibility: “The limitations of the study include the inability to collect attendance data at the Aussie-FIT weekly sessions and low retention at final follow up. One possible explanation for this loss could be the timing of data collections. Due to unavoidable time restrictions on when the program could be delivered, final follow ups were scheduled the week before Christmas, which is usually a busy time of personal commitments and festivities and may have prevented men from being able to attend. Unfortunately, we were unable to collect reliable data on men’s attendance at the program as coaches only sporadically recorded attendance. This was likely because coaches were busy at the start of the program, talking to men and preparing for the session. In a future trial, it may be more advantageous to use a ‘self-check in’ system on an iPad, where men can register when arriving at the venue. This study also lacked objective measurement of sleep quality, body composition and cardiorespiratory or neuromuscular fitness. A fully powered future trial could include these measurements to further improve quality of the outcome assessment. Also, some of the measured outcomes did not improve significantly in the intervention group at 3 months (e.g., waist circumference, blood pressure, sedentary time, need support). Nevertheless, findings were consistently in favour of the intervention group. To fully understand if there are no meaningful/significant differences, a fully powered trial needs to be conducted.  Another limitation of the current study is the generalisability of findings with respect to ethnicity as the sample was not representative of the Australian population (95% were Caucasian). Future formative work, including feedback on resources from a diverse sample of men. Future trials should investigate whether findings can be replicated in other ethnic groups, or whether the program requires additional tailoring to appeal to a more diverse sample of men in Australia (e.g., men from culturally and linguistically diverse backgrounds, indigenous men, men from across the socio-economic spectrum). As there are only a limited number of AFL clubs in Australia, a fully powered RCT may need to also rely on lower level clubs (e.g., state-based leagues) to reach target numbers and attract men from a wider geographical reach.” |  |
| Generalisability | 21 | Generalisability (applicability) of pilot trial methods and findings to future definitive trial and other: studies: “Another limitation of the current study is the generalisability of findings with respect to ethnicity as the sample was not representative of the Australian population (95% were Caucasian). Future formative work, including feedback on resources from a diverse sample of men. Future trials should investigate whether findings can be replicated in other ethnic groups, or whether the program requires additional tailoring to appeal to a more diverse sample of men in Australia (e.g., men from culturally and linguistically diverse backgrounds, indigenous men, men from across the socio-economic spectrum). As there are only a limited number of AFL clubs in Australia, a fully powered RCT may need to also rely on lower level clubs (e.g., state-based leagues) to reach target numbers and attract men from a wider geographical reach.” |  |
| Interpretation | 22 | Interpretation consistent with pilot trial objectives and findings, balancing potential benefits and harms, and considering other relevant evidence: “Our findings for weight loss at 3 months are comparable to most other gender-targeted interventions delivered in sports settings internationally. The results of our pilot study are comparable to those from the HockeyFIT pilot trial in Canada (57; *N* = 80) and the RuFIT pilot in New Zealand (16; *N* = 96), which reported mean differences in weight loss between groups at 3 months to be 3.6 kg (95% CI 1.9–5.3) and 2.5 kg (95% CI -0.4–5.4), respectively, in favour of the intervention. Compared to the FFIT (*N* = 748) trial, Aussie-FIT participants’ weight loss immediately post-program was lower; FFIT participants lost 5.18 kgs (−6.00 to −4.35) at 3 months [14]. However, any comparisons with FFIT must be interpreted with caution, given that the FFIT evaluation was not a pilot.  In relation to physical activity outcomes, we found only a small increase in step counts following participation in the program for the intervention arm, however, this warrants more rigorous testing via a fully powered RCT, given the relatively large proportion of participants with step counts meeting general PA guidelines at baseline. The FFIT study used a self-report measure of physical activity, whereas the recent EuroFIT intervention reported device-measured physical activity (steps) as the main outcome; ITT analyses in EuroFIT showed a baseline-adjusted mean difference of 1,208 steps per day (95% CI, 869–1,546) in favour of the intervention group at 3 months. It is difficult to explain the high baseline in step count in this study. Anecdotally, we are aware that many participants were in manual professions (e.g., construction work), hence, it is possible that there was a sampling bias, However, we did not collect data on occupations, so we cannot draw conclusions as to a potential bias. We also note that the ActiGraph algorithm classifies intermittent stepping more readily than the activPAL algorithm (used in the EuroFIT trial),which is shown to result in a non-trivial discrepancy between devices [66].  In line with other FIT studies [20, 67], Aussie-FIT participants successfully decreased their consumption of fatty and sugary foods following the program; point estimates indicated small increases in fruit and vegetable intake, however it is noteworthy that scores were already relatively high at the start of the intervention. Excessive alcohol consumption is often perceived to be central to the culture of footy fandom in Australia and the program specifically aimed to reduce it [68]. In the current sample, men reported low alcohol intake on average at baseline, which was below the alcohol guideline limits for Australian males. It is possible that men under-reported alcohol consumption, or that this study attracted a sample of men who were moderate drinkers.  The inclusion of motivational components in the coach training and program content of Aussie-FIT were a key adaptation and extension of previous trials. There is preliminary evidence to suggest that participating in the Aussie-FIT program led participants to experience increased feelings of autonomy, competence, and relatedness in relation to weight loss behaviours. These findings are important as, according to SDT [26], feeling that one is competent, autonomous, and related to others are critical determinants of adaptive behaviours, cognitions and emotions [69]. In a future RCT, longer term follow ups will help to determine whether participation in Aussie-FIT leads to longer term experience of need satisfaction and/or whether experiences of need support offered during Aussie-FIT are associated with better outcomes for participants.  We also assessed constructs that were targeted in the intervention as relevant for weight loss, but were not previously addressed in FIT-based studies. For instance, there was a significant improvement in goal facilitation. This tentatively suggests that Aussie-FIT participants learnt how to prioritise their weight loss. However, there was no meaningful effects for dealing with competing goals. This may be due to a low emphasis in the program content on dealing with other competing goals [70]. The improvements in self-reported behavioural automaticity for physical activity and healthy eating were consistent with findings from other studies promoting habit formation [71]. There was also improvement in planning at 3 months in favour of the intervention group, consistent with previous research on forming action and coping plans [55, 56].  Finally, there was improved self-reported sleep quality at 3 months in favour of the intervention group. Given the burgeoning concerns with the ill-health effects of bad quality of sleep [72], this result is promising. This finding is based on well-validated self-report instrument but warrants replication with device-measured monitoring of sleep quality. Other FIT studies did not examine sleep change as an outcome of the intervention. Sleep duration and quality have an important influence on physical activity levels and consequently on weight. Better sleep is correlated with higher physical activity and lower weight [73].  Our findings support progression to a fully powered RCT to further test the effectiveness of the Aussie-FIT program; a future trial should be powered with data from this pilot rather than the FFIT study, and that will also be powered to detect changes in secondary outcomes. The long-term goal of the Aussie-FIT program is to promote long-term maintenance of behaviour changes that have led to weight loss; therefore, longer-term follow-up studies are required to determine whether specific additions included in this program have an impact on long-term outcomes. For instance, FFIT program participants maintained intervention effects at 3.5 years post baseline [2.90 kg (95% CI 1.78, 4.02 kg), *p* < 0.001] [15]; further exploration is needed to assess if the inclusion of specific SDT techniques and BCTs adds value that corresponds with further weight reduction or weight maintenance at a desired level.  We intended to recruit coaches via the AFL clubs, however the clubs did not have coaches readily available to fulfil this role. As a result, three coaches were recruited via recommendations from the clubs and three coaches were independently identified by the research team. All Aussie-FIT coaches’ professional backgrounds (which included teaching, coaching, exercise instruction and sport science) meant they were likely to be equipped to create an environment that is seen as central to the success of FFIT, i.e., non-didactic, encouraging, interactive delivery style that incorporates appropriate banter to support vicarious learning and team spirit [74]. However, those coaches who were not directly associated with the club may have been less able to integrate into their coaching style other characteristics seen as contributing to FFIT’s success, such as the ‘behind the scenes’ stories and tacit knowledge of the inner workings of the club into program delivery [74]. To overcome this, within the coach training, the coaches were encouraged to think about how to incorporate the sport and club ‘feel’ within their delivery style. Based on qualitative data, the level of connection between the coach and the club did not seem to contribute to any variability in quality of experience any more than other relevant variables such as the coaches’ personality and motivation, or differences in facilities available to deliver the program. However, the extent to which the personal and situational characteristics that shape program delivery impact participants’ experiences of the program and observed outcomes could be further explored in future research, to help inform the most appropriate implementation model for Australia.  The preliminary cost effectiveness evaluation suggests that the Aussie-FIT programme was relatively inexpensive to deliver and potentially cost-effective, with estimated ICERs of $1315.21 per each additional man achieving a 5% weight loss and $39,756.42 per QALY gained after 3 months. Moreover, our calculated ICER per QALY lies within the commonly acceptable range considered to be good value for money in Australia [75, 76]. However, the reported ICER in this pilot study should be interpreted with caution; and may be overestimated since it does not include several relevant costs, such as direct medical costs and future health system costs due to the intervention. A complete and comprehensive cost effectiveness evaluation using the model piloted in this study is warranted in a fully powered trial.” |  |
|  | 22a | Implications for progression from pilot to future definitive trial, including any proposed amendments: “The pilot findings may be generalizable to delivery of the intervention in other professional sport settings in Australia, and broader implementation may be better achieved by capitalising on roll outs of the program in lower-level state-based leagues, and via adaptations to other sports. The Aussie-FIT model also has potential for expansion via delivery to specific population segments who are not necessarily overweight or obese, but insufficiently active to benefit health, and for whom existing interventions may not appeal (e.g., new dads, prostate cancer survivors). Pilot studies are needed to determine whether tailoring the program to engage these specific population groups can offer an alternative or superior opportunity to ‘usual care’. Overall, this study achieved the objectives of testing the feasibility of delivering and evaluating Aussie-FIT in the AFL context in Australia. Recommendations to use these methods to in a future definitive RCT are warranted. The Aussie-FIT program should be tested and implemented on a larger scale with maintenance effects tested over a longer timeframe (e.g., 1 and 2-year follow ups). “ |  |
| **Other information** | | |  |
| Registration | 23 | Registration number for pilot trial and name of trial registry: “**Trial registration:** Australian New Zealand Clinical Trials Registry: ACTRN12617000515392.” |  |
| Protocol | 24 | Where the pilot trial protocol can be accessed, if available: |  |
| Funding | 25 | Sources of funding and other support (such as supply of drugs), role of funders: “*Funding*  This research was supported by the Western Australian Health Promotion Foundation (Healthway), through research grant 31953.” |  |
|  | 26 | Ethical approval or approval by research review committee, confirmed with reference number: “*Ethics approval and consent to participate*  The Curtin University Human Research Ethics Committee (HREC2018-0458) provided ethical approval; all participants provided written informed consent before participation” |  |

Citation: Eldridge SM, Chan CL, Campbell MJ, Bond CM, Hopewell S, Thabane L, et al. CONSORT 2010 statement: extension to randomised pilot and feasibility trials. BMJ. 2016;355.

*We strongly recommend reading this statement in conjunction with the CONSORT 2010, extension to randomised pilot and feasibility trials, Explanation and Elaboration for important clarifications on all the items. If relevant, we also recommend reading CONSORT extensions for cluster randomised trials, non-inferiority and equivalence trials, non-pharmacological treatments, herbal interventions, and pragmatic trials. Additional extensions are forthcoming: for those and for up to date references relevant to this checklist, see [www.consort-statement.org](http://www.consort-statement.org/).

**S1B. The TIDieR (Template for Intervention Description and Replication) Checklist*:**

Information to include when describing an intervention and the location of the information

| **Item number** | **Item** | **Where located **** | |
| --- | --- | --- | --- |
|  |  | Primary paper | Other ^†^ (details) |
|  | **BRIEF NAME** |  |  |
| **1.** | Provide the name or a phrase that describes the intervention. | 1 | Aussie-FIT: a weight loss program in sport settings. |
|  | **WHY** |  |  |
| **2.** | Describe any rationale, theory, or goal of the elements essential to the intervention. | 5-7 | Described in the introduction section |
|  | **WHAT** |  |  |
| **3.** | Materials: Describe any physical or informational materials used in the intervention, including those provided to participants or used in intervention delivery or in training of intervention providers. Provide information on where the materials can be accessed (e.g. online appendix, URL). | 10-12 | Described in the methods section and: <https://www.eatforhealth.gov.au/guidelines/australian-guide-healthy-eating>  <https://livelighter.com.au/> |
| **4.** | Procedures: Describe each of the procedures, activities, and/or processes used in the intervention, including any enabling or support activities. | 10-12 | Described in the methods section and: https://bmjopen.bmj.com/content/8/10/e022663 |
|  | **WHO PROVIDED** |  |  |
| **5.** | For each category of intervention provider (e.g. psychologist, nursing assistant), describe their expertise, background and any specific training given. | 9-10 | “The intention was for all coaches to be recruited directly from the participating clubs. In reality, three coaches were recruited via the AFL clubs, and three additional coaches were identified by the research team, due to limited capacity within the clubs to provide additional coaches. The Aussie-FIT coaches had experience in coaching, and also professional experience in teaching, exercise instruction, or sport science. The coaches attended four half-day program delivery training workshops. These were delivered by two female members of the research team (EQ, DK) who had expertise in health psychology, motivation and behaviour change principles, and experience designing and delivering coach education workshops (EQ). “ |
|  | **HOW** |  |  |
| **6.** | Describe the modes of delivery (e.g. face-to-face or by some other mechanism, such as internet or telephone) of the intervention and whether it was provided individually or in a group. | 10 | Intervention provided face-to-face in the groups, details here: “The program was delivered at the two AFL clubs in Perth, Western Australia. There were two deliveries to groups of ~15 men at each club (on two different days of the week) in each group (i.e., intervention and control). In total there were four groups in the intervention arm, and four groups of men in the wait-list control group received the intervention at a later date.” |
|  | **WHERE** |  |  |
| **7.** | Describe the type(s) of location(s) where the intervention occurred, including any necessary infrastructure or relevant features. | 10 | “The program was delivered at the two AFL clubs in Perth, Western Australia. There were two deliveries to groups of ~15 men at each club (on two different days of the week) in each group (i.e., intervention and control). In total there were four groups in the intervention arm, and four groups of men in the wait-list control group received the intervention at a later date.” |
|  | **WHEN and HOW MUCH** |  |  |
| **8.** | Describe the number of times the intervention was delivered and over what period of time including the number of sessions, their schedule, and their duration, intensity or dose. | 10-11 | “Aligned with the FFIT program, the Aussie-FIT intervention included 12 weekly 90 minute sessions designed to promote physical activity, healthy eating, and weight loss. The program was delivered to groups of approximately 15 men by one coach and included classroom-based activities and physical activity sessions. In the early weeks, a little more than half of the session was dedicated to classroom activities, with less time allotted to the physical activity sessions. Over the course of 12 weeks, the balance shifted to expand the physical activity component to align with the men’s progress in fitness. The delivery style was informal, encouraging positive social interaction, humour, and ‘friendly banter’. The program was gender-sensitised with an emphasis on dietary and physical activity changes that are consistent with masculinised practices (e.g., ordering salad with a steak, getting back to being able to play football with children or grandchildren, discussion of the role of alcohol in weight loss, and fostering group support). The program supported participants to make small sustainable changes to their eating through portion control, reduced consumption of sugary drinks, energy-dense foods and alcohol, and a gradual increase in physical activity by choosing the activity that the men enjoy the most or could most easily incorporate into daily life. To make the program culturally appropriate, program content was adapted to reflect the Australian Guidelines for Healthy Eating [36], and resources from ‘LiveLighter’ (a healthy eating campaign in Australia) were built into the program to illustrate key principles (e.g., tool for ease of reading food labels). The program was designed to teach participants strategies for self-regulation, goal setting, and avoiding compensatory behaviours (e.g., overeating after intense physical activity), and to prevent relapse.” |
|  | **TAILORING** |  |  |
| **9.** | If the intervention was planned to be personalised, titrated or adapted, then describe what, why, when, and how. | 10-12 | https://bmjopen.bmj.com/content/8/10/e022663 |
|  | **MODIFICATIONS** |  |  |
| **10.^ǂ^** | If the intervention was modified during the course of the study, describe the changes (what, why, when, and how). | 23-24 | “We intended to recruit coaches via the AFL clubs, however the clubs did not have coaches readily available to fulfil this role. As a result, three coaches were recruited via recommendations from the clubs and three coaches were independently identified by the research team. All Aussie-FIT coaches’ professional backgrounds (which included teaching, coaching, exercise instruction and sport science) meant they were likely to be equipped to create an environment that is seen as central to the success of FFIT, i.e., non-didactic, encouraging, interactive delivery style that incorporates appropriate banter to support vicarious learning and team spirit [74]. However, those coaches who were not directly associated with the club may have been less able to integrate into their coaching style other characteristics seen as contributing to FFIT’s success, such as the ‘behind the scenes’ stories and tacit knowledge of the inner workings of the club into program delivery [74]. To overcome this, within the coach training, the coaches were encouraged to think about how to incorporate the sport and club ‘feel’ within their delivery style. Based on qualitative data, the level of connection between the coach and the club did not seem to contribute to any variability in quality of experience any more than other relevant variables such as the coaches’ personality and motivation, or differences in facilities available to deliver the program. However, the extent to which the personal and situational characteristics that shape program delivery impact participants’ experiences of the program and observed outcomes could be further explored in future research, to help inform the most appropriate implementation model for Australia.” |
|  | **HOW WELL** |  |  |
| **11.** | Planned: If intervention adherence or fidelity was assessed, describe how and by whom, and if any strategies were used to maintain or improve fidelity, describe them. | N/A | Planned intervention fidelity will be described in the process evaluation article. |
| **12.^ǂ^** | Actual: If intervention adherence or fidelity was assessed, describe the extent to which the intervention was delivered as planned. | N/A | Actual intervention fidelity will be described in the process evaluation article. |

** **Authors** - use N/A if an item is not applicable for the intervention being described. **Reviewers** – use ‘?’ if information about the element is not reported/not sufficiently reported.
