# Supplementary material for: A gender-sensitised weight-loss and healthy living program for men with overweight and obesity in Australian Football League settings (Aussie-FIT): A pilot randomised controlled trial
Source: PLoS Med. 2020 Aug 6;17(8):e1003136. doi: 10.1371/journal.pmed.1003136 (PMC7410214; doi:10.1371/journal.pmed.1003136)
Supplement: S2 Table — (DOCX) [file pmed.1003136.s002.docx]

*S2 Appendix.* Accelerometer data at all time points for participants with 4+ days of valid (i.e. >=10h/day) wear time. Data are means (SD).

|  | **Aussie-FIT intervention group** | **Aussie-FIT control group** | **Total** |
| --- | --- | --- | --- |
| **Time 1** | n=57 | n=64 | n=121 |
| Waking wear time (min/day) | 888.14 (92.90) | 878.53 (92.76) | 883.06 (92.56) |
| Sedentary (% of waking wear) | 66.52 (8.14) | 64.98 (8.82) | 65.705 (8.508) |
| Light activity (% of waking wear) | 29.45 (7.36) | 30.63 (8.00) | 30.075 (7.698) |
| MVPA (% of waking wear) | 4.03 (2.22) | 4.39 (2.55) | 4.22 (2.40) |
| Sedentary time (min/day) | 590.74 (94.55) | 571.9 (101.76) | 580.78 (98.47) |
| Light activity (min/day) | 261.80 (70.21) | 268.2 (71.7) | 265.20 (70.80) |
| MVPA (min/day) | 35.61 (19.57) | 38.38 (21.5) | 35.30 (19.21) |
| Uncensored step counts (n/day) | 12872 (3770) | 13097 (3754) | 12991 (3747) |
| Censored step count (n/day) | 10787 (3359) | 11053 (3557) | 10928 (3454) |
|  |  |  |  |
| **Time 2** | n=40 | n=52 | n=92 |
| Waking wear time (min/day) | 961.71 (202.18) | 958.33 (164.51) | 959.80 (180.83) |
| Sedentary (% of waking wear) | 66.93 (8.92) | 66.58 (9.57) | 66.74 (9.25) |
| Light activity (% of waking wear) | 28.24 (7.51) | 29.35 (8.37) | 28.87 (7.98) |
| MVPA (% of waking wear) | 4.83(2.59) | 4.06 (2.40) | 4.39 (2.50) |
| Sedentary time (min/day) | 650.84 (192.32) | 644.22 (171.81) | 647.10 (180.0) |
| Light activity (min/day) | 265.72 (67.83) | 276.48 (73.01) | 271.80 (70.62) |
| MVPA activity (min/day) | 45.144 (22.50) | 37.63 (20.4) | 40.901 (21.57) |
| Uncensored step counts (n/day) | 13717 (4322) | 13421 (4047) | 13550 (4148) |
| Censored step count (n/day) | 11654 (4018) | 11355 (3827) | 11485 (3892) |
| **Time 3** | n=30 | n=30 | n=60 |
| Waking wear time (min/day) | 853.58 (76.30) | 911.55 (92.87) | 882.57 (89.19) |
| Sedentary (% of waking wear) | 62.84 (10.06) | 61.83 (7.52) | 62.34(8.83) |
| Light activity (% of waking wear) | 32.50 (9.04) | 32.65 (7.46) | 32.56 (8.21) |
| MVPA (% of waking wear) | 4.67 (2.65) | 5.51 (3.06) | 5.09 (2.88) |
| Sedentary time (min/day) | 536.32 (96.12) | 561.96 (76.19) | 549.13 (86.96) |
| Light activity (min/day) | 277.36 (79.24) | 298.59 (75.82) | 287.98 (77.63) |
| MVPA activity (min/day) | 39.90 (22.49) | 51.00 (31.77) | 45.45 (27.86) |
| Uncensored step counts (n/day) | 13762 (4395) | 15736 (4620) | 14749 (4580) |
| Censored step count (n/day) | 11718 (4049) | 13438 (4406) | 12578 (4284) |
