# Supplementary material for: A gender-sensitised weight-loss and healthy living program for men with overweight and obesity in Australian Football League settings (Aussie-FIT): A pilot randomised controlled trial
Source: PLoS Med. 2020 Aug 6;17(8):e1003136. doi: 10.1371/journal.pmed.1003136 (PMC7410214; doi:10.1371/journal.pmed.1003136)
Supplement: S3 Table — (DOCX) [file pmed.1003136.s003.docx]

*S4 Appendix.* Changes from 3 months to 6 months for the intervention group only.

|  | **Per protocol (n= 47)** | **All cases analysis (n=64)** |
| --- | --- | --- |
| Weight (kg) | - .46 (-1.37, .44), *p* = .32 | - .98 (-2.60, .65), *p* = .24 |
| Waist circumference (cm) | -5.77 (-8.40, -3.13), *p* <.001 | -5.50 (-7.43, -3.57), *p* < .001 |
| BMI (kg/m^2^) | -.19 (-.48, .11), *p* = .22 | -.37 (-.88, .14), *p* = .16 |
| Blood pressure (mm/Hg)  Systolic  Diastolic | 2.44 (-1.82, 3.06), *p* <.001  1.11 (-2.52, 4.75), *p* = .55 | .51 (-1.53, 2.54), *p* = .62  .87 (-2.11, 3.84), *p* = .57 |
| Sedentary time | -91.14 (-191.75, 9.45), p = .08 (n=28) | -95.82 (-170.51, -21.12), p = .01 (n=35) |
| LPA time | 4.91 (-14.07, 23.90), p = .61 | 7.73 (-13.10, 28.56), p = .47 |
| MVPA time | -5.88 (-9.50, -2.27), p = .001 | -7.20 (-12.50, -1.91), p = .01 |
| Steps (uncensored) | -119.04 (-668.39, 430.30), p = .67 | 138.51 (-822.98, 1100.00), p = .78 |
| Steps (censored) | -106.25 (-676.02, 463.53), p = .72 | 55.4.0 (-904.36, 1015.15), p = .91 |
| DINE–based measures:  Fatty food score | .05 (-.01, .10), *p* = .10 | .02 (-.08, .11), *p* = .71 |
| Fruit and vegetable | -.04 (-.18, .11), *p* = .63 | -.01 (-.11, .08), *p* = .78 |
| Sugary food score | .06 (-.03, .14), *p* = .18 | .04 (-.06, .13), *p* = .44 |
| Total alcohol consumption | -2.58 (-4.26, -.90), *p* = .003 | -2.52 (-5.53, .49), *p* = .10 |
| Self–esteem Rosenberg scale | .02 (-.09, .13), *p* = .68 | .02 (-.10, .13), *p* = .76 |
| Positive affect (PANAS) | -.20 (-.30, -.10), *p* < .001 | -.17 (-.26, -.09), *p* < .001 |
| Negative affect (PANAS) | .08 (.01, .15), p = .03 | .08 (.01, .15), p = .03 |
| Need support in relation to weight loss (IBQ scale) | -.15 (-.52, .21), *p* = .40 | .07 (-.20, .34), *p* = .61 |
| Weight loss motivation:  Autonomous  Controlled | -.01 (-.20, .18), *p* = .88  -.05 (-.23, .14), *p* = .63 | .04 (-.18, .23), *p* = .72  -.07 (-.33, .19), *p* = .59 |
| Basic need satisfaction in relation to weight loss behaviours | -.24 (-.53, .05), *p* = .10 | -.12 (-.38, .14), *p* = .36 |
| Health-related quality of life (EQ-5) | -.03 (-.09, .03), *p* = .37 | -.01 (-.05, .04), *p* = .83 |
| Total for health today | 1.70 (-.43, 3.84), *p* = .12 | 2.59 (.42, 4.76), *p* = .02 |
| Goal facilitation  Competing goals | .04 (-.13, .20), *p* = .69  .01 (-.07, .09), *p* = .73 | .09 (-.05, .22), *p* = .21  -.02 (-.08, .04), *p* = .56 |
| Habits for physical activity  Habits for healthy eating | .07 (-.27, .41), *p* = .68  -.16 (-.43, .12), *p* = .26 | .16 (-.31, .63), *p* = .50  -.15 (-.46, .17), *p* = .35 |
| Barriers | -.18 (-.45, .08), *p* = .17 | -.04 (-.30, .21), *p* = .74 |
| Planning | -.37 (-.61, .12), *p* = .003 | -.26 (-.40, .12), *p* < .001 |
| Sleep quality (PSQI) | .60 (.28, .91), *p* < .001 | .34 (.01, .67), *p* = .046 |
