# Supplementary material for: A gender-sensitised weight-loss and healthy living program for men with overweight and obesity in Australian Football League settings (Aussie-FIT): A pilot randomised controlled trial
Source: PLoS Med. 2020 Aug 6;17(8):e1003136. doi: 10.1371/journal.pmed.1003136 (PMC7410214; doi:10.1371/journal.pmed.1003136)
Supplement: S4 Table — (DOCX) [file pmed.1003136.s004.docx]

*S5 Appendix.* Program effects (i.e., change from 3 months to 6 months) for the waitlist control group.

|  | **Per protocol (n=35)** | **All cases analysis (n= 66)** |
| --- | --- | --- |
| Weight (kg) | -3.55 (-4.68, -2.43), *p* < .001 | -3.43 (-4.40, -2.46), *p* < .001 |
| Waist circumference (cm) | -5.41 (-6.28, -4.54), *p* < .001 | -5.89 (-7.03, -4.74), *p* < .001 |
| BMI (kg/m^2^) | -1.11 (-1.48, -.74), *p* <.001 | -1.06 (-1.37, -.74), *p* <.001 |
| Blood pressure (mm/Hg)  Systolic  Diastolic | -.45 (-4.18, 3.29), *p* = .82  .29 (-2.58, 3.15), *p* = .85 | .32 (-3.43, 4.07), *p* = .87  .27 (-2.93, 3.47), *p* = .02 |
| Sedentary time | -97.14 (-172.60, -21.69), p = .01 (n=35) | -88.65 (-170.15, -7.16), p = .03 (n=41) |
| LPA time | 18.38 (-.78, 37.54), p = .06 | 19.50 (-4.04, 43.05), p = .10 |
| MVPA time | 7.45 (-.84, 15.74), p = .08 | 8.34 (-1.77, 18.44), p = .11 |
| Steps (uncensored) | 1359.87 (461.88, 2257.85), p = .003 | 1586.67 (844.87, 2328.46), p < .001 |
| Steps (censored) | 1233.64 (292.56, 2174.72), p = .01 | 1776.09 (1217.32, 2334.86), p < .001 |
| DINE–based measures:  Fatty food score | -.25 (-.32, -.18), *p* < .001 | -.28 (-.35, -.21), *p* < .001 |
| Fruit and vegetable | .14 (-.11, .38), *p* = .27 | .19 (-.002, .39), *p* = .052 |
| Sugary food score | -.33 (-56, -.09), *p* = .006 | -.33 (-56, -.10), *p* = .004 |
| Total alcohol consumption | -2.74 (-4.47, -1.01), *p* = .002 | -2.70 (-4.90, -.50), *p* = .016 |
| Self–esteem Rosenberg scale | .23 (.14, .33), *p* < .001 | .23 (.15, .29), *p* < .001 |
| Positive affect (PANAS) | .43 (.21, .66), *p* < .001 | .46 (.24, .69), *p* < .001 |
| Negative affect (PANAS) | -.16 (-.28, -.04), p = .007 | -.19 (-.35, -.04), p = .01 |
| Need support in relation to weight loss (IBQ scale) | .20 (-.03, .44), *p* = .09 | .22 (-.08, .52), *p* = .16 |
| Weight loss motivation:  Autonomous  Controlled | .19 (.05, .33), *p* = .008  -.20 (-.36, -.05), *p* = .012 | .19 (.06, .22), *p* = .003  -.24 (-.42, -.05), *p* = .012 |
| Basic need satisfaction in relation to weight loss behaviours | .49 (.31, .70), *p* < .001 | .54 (.39, .70), *p* < .001 |
| Health-related quality of life (EQ-5) | -.004 (-.06, .06), *p* = .91 | -.01 (-.07, .05), *p* = .81 |
| Total for health today | 9.27 (5.71, 12.83), *p* < .001 | 6.57 (1.45, 11.70), *p* = .01 |
| Goal facilitation  Competing goals | .44 (.30, .57), *p* < .001  -.52 (-.80, -.25), *p* < .001 | .44 (.32, .57), *p* < .001  -.51 (-.82, -.20), *p* = .001 |
| Habits for physical activity  Habits for healthy eating | .83 (.45, 1.20), *p* < .001  .71 (.33, 1.09), *p* < .001 | .73 (.39, 1.08), *p* < .001  .70 (.33, 1.07), *p* < .001 |
| Barriers | .17 (.02, .32), *p* = .03 | .15 (.05, .25), *p* = .04 |
| Planning | .44 (.31, .57), *p* < .001 | .36 (.26, .45), *p* < .001 |
| Sleep quality (PSQI) | -1.20 (-1.64, -.76), *p* < .001 | -1.40 (-1.82, -.98), *p* < .001 |
