# Supplementary material for: A gender-sensitised weight-loss and healthy living program for men with overweight and obesity in Australian Football League settings (Aussie-FIT): A pilot randomised controlled trial
Source: PLoS Med. 2020 Aug 6;17(8):e1003136. doi: 10.1371/journal.pmed.1003136 (PMC7410214; doi:10.1371/journal.pmed.1003136)
Supplement: S1 Text — (SPSS file, here: https://osf.io/4vsng/files/). (DOCX) [file pmed.1003136.s005.docx]

*Supplement 3.* All scales reliability checks. (SPSS file, here: <https://osf.io/4vsng/files/>)
