# Supplementary material for: A gender-sensitised weight-loss and healthy living program for men with overweight and obesity in Australian Football League settings (Aussie-FIT): A pilot randomised controlled trial
Source: PLoS Med. 2020 Aug 6;17(8):e1003136. doi: 10.1371/journal.pmed.1003136 (PMC7410214; doi:10.1371/journal.pmed.1003136)
Supplement: Attachment — Submitted filename: R1 response_Plos Medicine_AussieFIT_16.03.2020_FINAL.docx [file pmed.1003136.s006.docx]

**Response to the comments for "A gender-sensitised weight loss and healthy living program for overweight and obese men in Australian Football League settings (Aussie-FIT): A pilot randomised controlled trial." (PMEDICINE-D-19-03524) for consideration at PLOS Medicine**

We would like to thank you for the feedback on our manuscript received on 25^th^ February 2020. We are pleased to have the opportunity to revise and resubmit manuscript D-19-03524 to *Plos Medicine*. We would like to thank you and the three reviewers for their positive comments regarding our submission, and express our appreciation for the suggestions offered to help improve the quality of our manuscript. We have provided a point-by-point response to all comments, indicating all changes that have been made to the manuscript, or a rationale as to why we have not taken action. Any words, sentences, or sections that have been inserted/revised from the previous resubmission are clearly displayed in our revised manuscript using tracked changes. The page and line numbers referenced in this response align with our marked up manuscript. Any citations that we provide in this letter, which are not included in our manuscript, are presented in full at the end of this response.

Sincerely,

Authors

**Requests from the editors:**
 *1. Abstract- please use an alternative to “hook”*

**Response:** We have replaced the word “hook” with “draw”, page 2, line 27.

*2. Abstract- please use specific language instead of “meaningful differences”. If you choose to report your secondary outcomes here, we suggest you include 95% CI and p values.*

**Response:** We have decided to omit the sentence: “Meaningful differences were observed in several secondary outcomes favouring the intervention group”. This omission will ensure that the abstract stays within the journal’s word constraints, page 2, line 47.

*3. Abstract methods and findings- the last sentence of this section should highlight a limitation of your study design*

**Response:** We have revised the end of the conclusion to highlight a limitation of the study design: “Issues with retention were a limitation of this trial. In a future, fully powered randomised controlled trial (RCT), retention could be improved by conducting assessments outside of holiday seasons.” Pages 2-3, lines 49-57.

*4. At this stage, we ask that you include a short, non-technical Author Summary of your research to make findings accessible to a wide audience that includes both scientists and non-scientists. The Author Summary should immediately follow the Abstract in your revised manuscript. This text is subject to editorial change and should be distinct from the scientific abstract. Please see our author guidelines for more information:* [*https://journals.plos.org/plosmedicine/s/revising-your-manuscript#loc-author-summary*](https://journals.plos.org/plosmedicine/s/revising-your-manuscript#loc-author-summary)

**Response:** We have added a non-technical Author Summary (see page 3 and 4).

“**Why Was This Study Done?**

- The prevalence of overweight and obesity is higher in men than in women in Australia (71% versus 56%).
- Provision of weight loss programs suitable for men is limited, and men are less likely than women to take part in such programs.
- Use of sport settings, such as Australian Football League (AFL) club facilities, can be used as a powerful way to encourage men to participate in weight loss programs.

**What Did the Researchers Do and Find?**

- 130 men took part in the Aussie-FIT program. This men-only weight loss program included 12 weekly 90-minutes sessions that included weight loss education and coach-led physical activity. The AFL themed program was delivered in two Australian football club settings.
- After all of the men’s initial weight and health measures were taken, half of the group received the program and the other half waited for three months to receive it. At 3 months, the measurements were repeated so that we could compare differences in weight (and other indicators of health) between men who had already completed the program and the men who were waiting to receive the program.
- We found that the men who had participated in the program lost on average 3.33kg more than the men who had not. Men who had completed Aussie-FIT did, on average, 8.54 minutes more moderate-to vigorous physical activity per day than their counterparts who had not yet received the program.

**What Do These Findings Mean?**

- Aussie-FIT is feasible to deliver in Australian football settings. This study provides some initial evidence that Aussie-FIT may be appealing and effective as a weight loss program for overweight and obese men in Australia.
- This study was relatively small (130 men took part). In order to make definitive policy recommendations regarding program scale out, a follow up study involving more participants and more football clubs is needed.
- A limitation of this study was that the program did not attract men from culturally or linguistically diverse backgrounds or indigenous Australians; future studies should explore how the program can be made more appealing and acceptable to men from more diverse backgrounds.’’

*5. Please include the study protocol document and analysis plan, with any amendments, as Supporting Information to be published with the manuscript if accepted. If this is published, please provide clear details in the methods section.*

**Response:** The study protocol is reported in the protocol paper (Quested et al., 2018) and was pre-registered with the Australian New Zealand Clinical Trials Registry (ACTRN12617000515392) where amendments are documented. Both these sources of information are open access. Providing the same information in a supplementary material will duplicate information. The published protocol is referred to and referenced in the methods section. We have updated this sentence to clarify that amendments to the protocol are documented with the registry: “The study protocol has been published elsewhere [22]” (page 7, line 170-171). The only amendment to the protocol was the recruitment and training of 6 coaches, not 4, as was stated in the protocol; this amendment is detailed in the records of the Australian New Zealand Clinical Trials Registry (ACTRN12617000515392)”.

*6. References must be within square brackets throughout*

**Response:** We have changed the references so they are in square brackets throughout.

*7. Please provide the primary and secondary outcome measures within the methods section. A brief summary of the trial outcomes can be presented in the introduction section*

**Response:** We have reduced the amount of detail provided about trial outcomes in the introduction (page 7), and have listed the primary and secondary outcome measures in the methods section (page 13, Measures -1^st^ paragraph): “The primary outcome in this trial was mean difference in weight between groups at 3 months, adjusted for baseline weight. Secondary outcome measures were feasibility of recruitment and trial retention, device-measured weight, waist and blood pressure, physical activity, self-reported diet and alcohol, well-being, quality of life, motivation for physical activity and other variables relevant to behaviour change, and self-reported sleep.”

*8. Questionnaires -please provide the questionnaires used in this study as SI files or if previously published, please provide a citation*

**Response:** All questionnaires used within this study have been published elsewhere, and the citations are provided in the reference list. Further details on questionnaires are also provided in the study protocol paper (Quested et al., 2018).

*9. Please provide 95% confidence intervals along with all p-values, all p-values should be in 0.xx format*

**Response:** We have provided 95% confidence intervals along with all p-values, all p-values are now in 0.xx format. We have followed the APA guidelines for presenting p-values. According to the guide p < .001 is appropriate for p values smaller than that number. “When reporting p values, report exact p values (e.g., p = .031) to two or three decimal places. However, report p values less than .001 as p < .001. The tradition of reporting p values in the form p < .10, p < .05, p < .01, and so forth, was appropriate in a time when only limited tables of critical values were available” (p. 114).

*10. Discussion- I recommend that the results outlined at line 448 are reported in the results section*

**Response:** We deleted the results outlined at line 448 and now they are only reported in the results section.

*11. The primary outcomes on your trial registry do not fully match the primary outcomes reported in the manuscript, For instance- acceptability to participants and coaches. If a clarification is needed in the submission, please revise.*

**Response:** Since our original submission we have updated the trial registry to clarify that differences in weight loss between groups at 3 months was the primary outcome. All other outcomes were secondary. Some secondary outcomes (e.g., acceptability to participants and coaches) will not be reported in this paper, but the study process evaluation paper, which is in progress.

*12. Please include sections and paragraph numbers instead of page numbers in the CONSORT checklist*

**Response:** We were unsure about whether to make changes based on this comment, as we consulted the CONSORT guidelines and followed the recommendations to use page and line numbers. We have updated these in S1 to align with the ‘marked up’ version of this revised manuscript. (Note. If viewing with a Mac, these will also align with the clean version in Word as well).

*13. The Data Availability Statement (DAS) requires revision. For each data source used in your study:
a) If the data are freely or publicly available, note this and state the location of the data: within the paper, in Supporting Information files, or in a public repository (include the DOI or accession number).
b) If the data are owned by a third party but freely available upon request, please note this and state the owner of the data set and contact information for data requests (web or email address). Note that a study author cannot be the contact person for the data.
c) If the data are not freely available, please describe briefly the ethical, legal, or contractual restriction that prevents you from sharing it. Please also include an appropriate contact (web or email address) for inquiries (again, this cannot be a study author).*

**Response:** The data are not freely available, as we did not have participant consent to store de-identified data in an online depository. Therefore, option C applies. Enquiries can be directed to the Curtin University Human Research Ethics Committee ([hrec@curtin.edu.au](mailto:hrec@curtin.edu.au)). We have revised the data availability statement accordingly, pages 31-32.

**Comments from the reviewers:**

**Reviewer #1**

*This manuscript reports the results from a pilot of the Aussie-FIT program. The manuscript is well written and descriptive and I only have a couple of minor comments.*

**Response:** We thank reviewer 1 for these positive comments about our manuscript. *1. Line 105: Please change to "...healthy habits to maintain long-term behaviour change."*

**Response:** Thank you for this suggestion, we have changed the wording accordingly, page 6.

*2. Lines 115-124: You do not need to list all the outcomes here - just a brief summary is sufficient for the introduction*.

**Response:** We have revised this section to include only a brief summary of outcomes, thank you.

*3. Lines 173-175: Was feedback on the program only from 5 men? This is very small, and I would consider adding to the discussion that more formative work may have helped make the program more culturally appropriate for Australian men.*

**Response:** Feedback on the Aussie-FIT participant book was obtained from five men. We have added a comment to the discussion section that highlights the importance of additional formative work as important for the future generalisability of the findings to men in Australia: “Another limitation of the current study is the generalisability of findings with respect to ethnicity as the sample was not representative of the Australian population (95% were Caucasian). Future formative work, including feedback on resources from a diverse sample of men. Future trials should investigate whether findings can be replicated in other ethnic groups, or whether the program requires additional tailoring to appeal to a more diverse sample of men in Australia (e.g., men from culturally and linguistically diverse backgrounds, indigenous men, men from across the socio-economic spectrum)”. page 27.

*4. Line 181: Please state who delivered the training workshops.*

**Response:** We have added: “The coaches attended four half-day program delivery training workshops. These were delivered by two female members of the research team (EQ, DK) who had expertise in health psychology, motivation and behaviour change principles, and experience designing and delivering coach education workshops (EQ).” page 9.

*5. Lines 349-350: Please add the proportions of men (i.e. in %) for follow-up measures.*

**Response:** We have added the proportions of men (i.e. in %) for follow-up measures, page 17.

*6. Lines 372-373: When reporting the results, please provide more information as it is unclear exactly what you are measuring here i.e. what does the 2.88% refer to? I see this is clearer in the table but still needs to be written out in full in the text.*

**Response:** We have edited the text accordingly: “The mean difference at 3 months between groups was 3.33 kg (95% CI 1.89-4.77) or 2.88% of total body weight lost (1.48-4.28) from baseline, both in favour of the intervention arm (p < 0.001) as per all cases analysis (N=130); see Table 2.” Page 18.

*7. Line 390: Are you referring to sleep quality here, because as it stands I initially interpreted it as sleep duration (in which case decreasing sleep duration would not be a positive outcome!) Given that the sentence opens with "...reported significantly higher levels of..." this still refers to the sleep outcome (which decreased). This is very confusing. To aid understanding, I suggest including the sleep quality measure as its own sentence i.e. Sleep quality improved from baseline to a greater extent in the intervention versus control group. And I would update the table reporting sleep outcomes to indicate the a lower score indicates a better outcome.*

**Response:** Thank you for this suggestion, we have edited the text accordingly and added the sentence “Sleep quality improved from baseline to a greater extent in the intervention than in the control group (-1.18; 95% CI -2.16, -0.20, p = .02)” page18. The table has also been updated.

*8. Discussion: I think you need to discuss the generalisability of findings with respect to ethnicity - given that over 95% of participants were Caucasian, the sample isn't representative of the Australian population - and future work would need to investigate whether findings can be replicated in other groups - formative work may be needed to establish how the program could be tailored appropriately.*

**Response:** Thank you for this suggestion, we fully agree that the sample isn't representative of the Australian population. We have further elaborated on this point in the discussion, and suggested that future work would need to investigate whether findings can be replicated in other groups and that formative work may be needed to establish how the program could be tailored appropriately: “Another limitation of the current study is the generalisability of findings with respect to ethnicity as the sample was not representative of the Australian population (95% were Caucasian). Future formative work should investigate whether findings can be replicated in other ethnic groups, or whether the program requires additional tailoring to appeal to a more diverse sample of men in Australia (e.g., men from culturally and linguistically diverse backgrounds, indigenous men, men from across the socio-economic spectrum).” Page 25, lines 684-690.

**Reviewer #2: Colin Greaves review: Accept with Minor Revision**

*Overall, this is a very clearly written and high-quality report of an important pilot study around the adaptation /piloting of the highly successful FFIT intervention for implementation in an Aussie Rules context in Western Australia.*

**Response:** Thank you for your positive feedback on the importance and quality of our study.

*I have only some minor, mainly presentational comments (and a more substantive comment about health economics, which is made based on exposure to health economic evaluations in several full-scale trials, although I am not an expert in this area)*

*1. Abstract: Could mention 3-6 mth results (see below comments on presentation of these). One decimal place might be sufficiently precise?*

**Response:** We have not included 3-6 months results due to the abstract word limit. We have reported 2 decimal places as per APA guidelines throughout the manuscript.
 *2. BACKGROUND:
"Men need access to plentiful resources to maintain their behaviour long term, including both physical (access to healthy food, exercise 108 equipment) and psychological 109 (energy levels, positive mood) resources to facilitate health behaviour change maintenance 110 (28,29)."  ... and social resources?*

**Response:** This is a very good point. We have added “and social resources” to this sentence, page 7, line 152.

*METHODS
Sample size:
3. "The study sample size followed guidelines for pilot RCTs (30,31) and approximated the FFIT pilot study sample size (32). The Aussie-FIT pilot study was designed to inform the development of a definitive future trial.
- Given the additional /non-standard pilot study aim to 'assess preliminary efficacy', you should present a formal sample size calculation (based on detecting a difference in weight loss). It is not sufficient to simply refer to other pilot studies as a justification for the sample size selected. Even if the aims were only to estimate retention rates, a sample size calculation can be presented (e.g. calculate N needed to estimate retention rates with CIs of +/- 8%)*

**Response:** In the revised manuscript, to add context to interpretation of our findings, we report sample size calculations for detecting a difference in weight loss, as per the reviewer’s suggestion (see first sentence). We now report in the manuscript: “To add context to the findings, we also ran power analyses using data from the FFIT trial [14]. In terms of mean difference at 12 weeks of 4.71% (SD of difference=3.95), estimates of power =.9, alpha=.05, cluster size =15, 20% dropout, and ICC=.05, a sample size of 64 participants per group was required (total n=128).” We have also included a power calculation for retention rates: “With a sample size of 130, we will be able to estimate a drop-out rate of 20% to within a 95% confidence interval of +/- 7%” (page 8, lines 193-194).

We have also added a sentence to the discussion to clarify that we are recommending ‘a future trial should be powered with data from this pilot rather than the FFIT study, and that will also be powered to detect changes in secondary outcomes’, page 23, lines 632-634.

*4. Maybe say something about the background experience /qualifications of the facilitators? (in Discussion, how does this compare with FFIT and how might this have affected performance /interactions with participants?)*

**Response:** The Aussie-FIT coaches had experience in coaching, and also teaching, exercise instruction or sport science. We have added these details to the method (page 9, lines 232-233): “The Aussie-FIT coaches had experience in coaching, and also professional experience in teaching, exercise instruction, or sport science”. We have added a section to the discussion that reflects on how any differences between the coaches in Aussie-FIT and in FFIT may have impacted interactions with participants: “We intended to recruit coaches via the AFL clubs, however the clubs did not have coaches readily available to fulfil this role. As a result, three coaches were recruited via recommendations from the clubs and three coaches were independently identified by the research team. All Aussie-FIT coaches’ professional backgrounds (which included teaching, coaching, exercise instruction and sport science) meant they were likely to be equipped to create an environment that is seen as central to the success of FFIT, i.e., non-didactic, encouraging, interactive delivery style that incorporates appropriate banter to support vicarious learning and team spirit [74]. However, those coaches who were not directly associated with the club may have been less able to integrate into their coaching style other characteristics seen as contributing to FFIT’s success, such as the ‘behind the scenes’ stories and tacit knowledge of the inner workings of the club into program delivery [74]. To overcome this, within the coach training, the coaches were encouraged to think about how to incorporate the sport and club ‘feel’ within their delivery style. Based on qualitative data, the level of connection between the coach and the club did not seem to contribute to any variability in quality of experience any more than other relevant variables such as the coaches’ personality and motivation, or differences in facilities available to deliver the program. However, the extent to which the personal and situational characteristics that shape program delivery impact participants’ experiences of the program and observed outcomes could be further explored in future research, to help inform the most appropriate implementation model for Australia.” (page 23-24, lines 633-652).

*5. Intervention: what content /contact (if any) beyond 12 weeks? important to know given the emphasis on maintenance here.*

**Response:** We have added information regarding contact beyond 12 weeks: “There was no formally structured intervention content or contact provided after 12 weeks. Participants were free to communicate through the Facebook group with the coach and with each other. We encouraged the participants to keep in touch and to schedule to meet and to exercise together after the program had finished.” Page 12, lines 295-298.

*6. "We measured health-related quality of life using the EuroQol five-dimensional five level version (EQ-5D-5L, range 1-5), with lower scores indicative of higher health-related quality of life." - How was a single /overall QoL score computed?*

**Response:** This is an average of 5 components/domains (1-5 range), with lower scores indicative of higher health-related quality of life. We added relevant info to the article: “We measured health-related quality of life using the EuroQol five-dimensional five level version (EQ-5D-5L, range 1-5, mean score), with lower scores indicative of higher health-related quality of life [53] and EQ-5 overall score (range 0-100), where high scores indicate better overall health.” Page 15, lines 374-378.

*7. Analysis: How were missing data imputed for the ITT analysis? e.g. baseline carried forward, LOCF?*

**Response:** We used full information maximum likelihood estimation to handle missing data, which is consistent with recommendations for the treatment of missing data in RCTs (White et al., 2011). We added: “We conducted an all-cases analysis that involved all participants who were randomised to the intervention or control group and provided at least baseline data; missing data were handled using full information maximum likelihood estimation [62].” Page 16, lines 392-395.

*8. I am a bit confused by the logic behind the secondary analysis which seems to combine "maintenance effects for the intervention group and treatment effects for the control group". So, by combining, it measures neither of these things? It might be better to present the 3-6 month within-group changes separately? Not as a comparison - just mean differences with 95%CIs?*

**Response:** We have updated the manuscript to present these results separately: supplementary material 4 shows maintenance effects for the intervention group only and supplementary material 5 shows post-intervention effects for the control group only. We have also edited the methods section to clarify that these were two separate within group analyses, not between group analyses. We have included results from these analyses as supplementary materials, but, as they do not contribute directly to the goals of this pilot and are not appropriately powered to be conclusive, we have not described these findings in the results, or discussed them in the discussion. Page 19, lines 468-469: “Intervention (S4 Appendix) and control (S5 Appendix) group effects were analysed separately for the 3 and 6 months data.”

*9. I also don't follow the following "We modelled the linear effect of time (3 to 6 months) as a predictor of dependent variables, adjusted for baseline values and the clustering effect of group. In so doing, the effect can be interpreted as the average amount of change on the dependent variable between 3 and 6 months." I'm not a statistician, but I cant see how that interpretation arises. It might be better just to lose this analysis (as above)?*

**Response:** The secondary analyses were executed on the intervention or control groups in isolation, so that we could explore maintenance and treatment effects in each group separately. To do so, we created a time variable where 3 months was coded as “0” and 6 months was coded as “1”. The dependent variable is regressed on this time variable, which represents a linear effect based on the coding approach, and the baseline score for the dependent variable. In essence, this variable enables us to capture within-person change from 3 to 6 months, which is adjusted for the baseline score on the dependent variable. The paper has been updated to make clear that the secondary analyses were executed on each group separately (see page 16). As described in response to comment #8, these results are not included in the main body of the manuscript. The text now is: “Secondary analyses focused on maintenance effects for the intervention group and treatment effects for the control group; these analyses were conducted on each group separately. In separate, within-group analyses, we modelled the linear effect of time (3 to 6 months) as a predictor of dependent variables, adjusted for baseline values and the clustering effect of group. In so doing, the effect can be interpreted as the average amount of change on the dependent variable between 3 and 6 months.” (page 16, lines 397-402).

*RESULTS:
10. Baseline steps and MVPA are high? It is a bit hard to say anything definitive about MVPA as unbouted MVPA is usually 5-10 times higher than MVPA in ten-minute bouts, depending on the sample. Steps do look high though, suggesting there may be some sample bias here - so it may be worth picking this up in the limitations - and say how you might seek to ameliorate this in the full-scale trial?*

**Response:** We agree that the participants’ baseline steps were higher than expected for an overweight/obese population, and also higher than were reported in the recently published Euro-FIT trial (Wyke et al., 2019). It is difficult to explain this high baseline in our study, based on the data collected as we did not collect information on the participants’ occupations, or other demographic information that may account for this. Anecdotally, we are aware that many participants were in manual professions (e.g., construction work), hence, it is possible that there was a sample bias, however we did not collect data on occupations so we cannot draw conclusions as to a potential bias. We have added this limitation to the discussion section and have recommended that occupation is recorded in future trials (page 23, lines 541-547). We also note that the ActiGraph algorithm classifies intermittent stepping more readily than the activPAL algorithm (used in the EuroFIT trial, Wyke et al., 2019) which is shown to result in a non-trivial discrepancy between devices (Steeves et al., 2015).

*11. 3 month outcomes: Please make clear that the analyses presented in the first and second paragraphs (3.33 kg (95% CI 1.89-4.77) etc) are the ITT (all participants with imputed values) figures. You could also summarise the per protocol data here?*

**Response:** We have added ‘all cases analysis’ to the section headings to improve clarity. The change in phrase to describe our analysis from ‘intention to treat’ to ‘all cases analysis’ is explained in response to comment 1 of Reviewer 3. Results for the completers are all listed in the tables.

*12. Is "overall health the EQ-5D-5L score (and if so, which one - the thermometer or other summary score)?*

**Response:** We added that this is “overall health as per EQ-Visual Analogue Scale (14.57; 95% CI 9.77, 19.34, p < .001)”. This is a 0-100 rating.” (page 18, lines 462-463).

*13. 3-6 month outcomes: As above, the rationale for this analysis does not make sense - it combines initial weight loss in the control group with post-intervention maintenance in the intervention group and uses a rather odd (to me) statistical approach (estimating 3-6 month change for the combined groups, whilst controlling for baseline values). Why not simply report the 3-6 month changes within each group (mean change = ***kg, 95%CI: *** to ***). The best estimate of 3-6 maintenance would be the within intervention group change from 3 to 6 months, where weight appears to further decrease by around 0.6kg (Table 2). Also need to report this figure with and without imputation of missing values.*

**Response:** Apologies for the miscommunication. We actually executed the secondary analyses on the experimental groups separately, so that we could explore maintenance effects in the intervention group (3-6 months) and treatment effects in the control group. So, our approach is consistent with your recommendation in that it provides a statistical test of the within group change in dependent variables from 3-6 months, adjusted for baseline scores. The raw or unadjusted scores are provided in Table 2. We have updated the manuscript so that it is clear these analyses were conducted separately on the experimental groups (page 16). As noted above in our response to one of your other comments (#7), we used full information maximum likelihood estimation to handle missing data which uses all available data rather than imputes data. We performed all secondary analyses using an all cases approach; this point has been clarified in the revised submission (page 16, lines 393-395).

*14. Cost-effectiveness: The calculations here seem to be more back-of-the-envelope than based on professional health economic analytic methods? I am not a health economist, but I do know that a) you have to include health-care usage and other societal costs (depending on the perspective taken) in the "cost" side of the equation (for both groups) and b) you don't just subtract utility values and divide the cost by this difference - there is a statistical analysis of individual level data involved which yields 95%CIs around the estimate. NB: I wouldn't expect to see a good ICER for within-trial analysis of EQ-5D data in a weight loss trial. The sensitivity of EQ-5D is too low and the main QoL benefits (not getting diabetes, avoiding CV events, cancer etc) that will actually influence EQ-5D tend to accrue in the longer term. A decision-analytic modelling approach would therefore be more appropriate for the main trial.*

**Response:** From a health economics perspective, our goal in this project was to develop and pilot an economic model to test procedures and measures that could be used to estimate cost-effectiveness of the program in a future full-scale trial. Preliminary cost effectiveness analyses can be useful for this purpose (e.g., Merlin et al., 2019). As alluded to in comment 15, it was not appropriate to conduct a comprehensive economic analysis based on the sample size in this pilot. We therefore did not collect data such as health-care usage in this study. For the purposes of the pilot, we were primarily interested feasibility of collecting the data and in program costs. Nevertheless, to test the economic model, procedures and measures, as well as to offer some context regarding the potential economic benefits from this program, we reported incremental cost effectiveness based on the pilot data. We have revised the manuscript throughout to emphasise that this was preliminary cost effectiveness for the purposes of informing future cost effectiveness analysis, and to emphasise that a more comprehensive cost effectiveness is warranted.

*15. Furthermore, it doesn't make sense to try to construct a full-blown health economic analysis based on only 110 participants and a 3-month timeframe. I would remove this health economic analysis and just report intervention costs, any between group difference in EQ-5D at 3 months (as part of Table 2) and the measures-completion rate - which indicates whether or not a health economic analysis would be feasible for the main trial.*

**Response:** We agree that our findings should not be interpreted as a comprehensive cost evaluation, however we have retained the ICER findings based on the justification in our response to point 14. We have also revised our manuscript accordingly to incorporate a caveat to emphasise: “the reported ICER in this pilot study may be an overestimate since it does not include several relevant costs such as direct medical costs and future health system costs due to the intervention, etc” (page 24, lines 657-660).

*16. Similarly, I would remove the probabilistic sensitivity analysis - the data is much too thin (low N) to bear this type of analysis?*

**Response:** We have removed the sensitivity analysis method, results and references to these analyses in the discussion.

*DISCUSSION

17. This is quiet long /rambling. It could benefit form some sub-headings to structure the text: Summary of findings; Relationship to other literature, Strengths and Limitations; Future research directions; Conclusion*

**Response:** We have added the suggested headings and re-structured some parts of the discussion to improve readability. We have edited some parts of the discussion to be more succinct.

*18. I would cut /substantially edit the health economics text (as per comments above).*

**Response:** We have substantially edited this section.

TABLES

*19. In general, please consider the appropriate number of significant figures /decimal places. Are your estimates really so accurate as to justify reporting of age, weight etc to 2 decimal places?*

**Response:** We followed the journal style and APA guidelines in the presentation of decimal places.

*20. Table 2: Are the means reported at 3 and 6 months imputed means (for all 64/66 participants) or per protocol means (for responders only) - please indicate.*

**Response:** Thank you for this suggestion, we have added that group means are completers’ means.

*21. Overall, this is a very worthy paper and a pleasure to read. Good luck with the revisions.*

**Response:** Thank you for your insightful and complimentary comments about our paper. We much appreciate your feedback and your suggestions for improvements.

**Reviewer #3: Alex McConnachie, Statistical Review**

*Kwasnicka and colleagues present a report of a pilot RCT of a weight loss and healthy lifestyle intervention delivered in Australian Football League clubs. This review considers the statistical aspects of the paper.

1. I note that the analysis is described as intention to treat, including everyone who provided baseline data. It is stated that ITT analysis "was used to deal with missing data". In my opinion, ITT analysis has nothing to do with missing data. As the name suggests, I would say that ITT is about which intervention it was intended to deliver. In other words, ITT means to analyse according to randomised group, regardless of whether the intervention was received or not. Missing data is a separate issue. Given the description of the statistical methods, I assume that the analysis involved analysing data from all time points, including baseline, in a mixed effects regression model. This accounts for missing follow-up data to some extent, and is a perfectly reasonable way to carry out the analysis. My only concern is the description of this being "an ITT analysis"; I would separate the methods used to deal with missing data from the concept of ITT.*

**Response:** Thank you for spotting this narrative error. We conducted an “all cases analysis” in which we included all participants who were randomised to the intervention or control group and provided at least baseline data, and compared these results with a per protocol analysis. We have updated the manuscript to clarify this point: “We conducted an all-cases analysis that involved all participants who were randomised to the intervention or control group and provided at least baseline data; missing data were handled using full information maximum likelihood estimation [62]. (page 16, lines 392-395).

*2. The 5 items of the EQ-5D questionnaire appear to have been averaged to give a score between 1 and 5. This is not usual; the responses to the 5 questions should be mapped to a health utility score at each time point. It is these health utilities that are used to derive QALYs.*

**Response:** In the data used in economic analysis, we had done exactly what Reviewer 3 describes. That is, EQ-5D-5L values were converted into utility weights on the basis of the preference weights of a pilot sample from the Australian general population (Norman, Cronin, & Viney, 2013) to compute QALYs. At 3 months, the average QALYs for the intervention group were 0.216 (SD=0.041) while for the comparison group it was 0.209 (SD=0.037). The incremental QALY gains at 3 months were estimated to be 0.007 (i.e. 0.216-0.209). We have revised the methods to clarify that the converted data to create QALYs were used in the economic analyses, whereas quality of life scores were used in the main analyses.

*3. Some of the data has been reported over-precisely. E.g. from line 355 onwards, we have summaries of a number of measures (weight, BP, MVPA) reported to 2 decimal places.*

**Response:** We have used two decimal places, as this is the requirement of the journal.

*4. Perhaps a little too much emphasis is put onto the statistical significance (or lack of) for between-group comparisons of some of the outcomes. More emphasis should be put on the point estimates of intervention effects (and their CIs). As the authors note at one point, in a pilot trial, the power will be limited to detect important differences between groups. It appears that the majority of measures have point estimates for the between-group difference at 3 months that are in favour of the intervention group. Some of the estimates (e.g. waist circumference, estimated mean difference -2.66cm) are quite sizeable, so whilst they are not statistically significant, I would say that these estimates add to the overall picture of clinically important intervention effects.*

**Response:** We agree with this point, and have revised reference to statistical significance throughout the discussion section.

*5. Similar comments apply to the changes between 3 and 6 months - a point estimate of an additional 0.98kg weight loss after the end of the intervention is more than just a lack of weight regain - to me, this suggests that the 3-month intervention may continue to have beneficial effects after the end of intervention delivery. This is what would be hoped of a lifestyle intervention, and though the change was not statistically significant in this pilot trial, it is promising, and lends support to the plan to do a larger trial.*

**Response:** In response to feedback from the other reviewers, we have now removed reference to results between 3 and 6 months in the results section, so this sentence is no longer in the manuscript.

*6. This may be a matter for the journal editors, but I prefer to read "0.xx", rather than ".xx".*

**Response:** We have changed this throughout, thank you.

**References cited in this response:**

Hunt, K., Wyke, S., Gray, C. M., Anderson, A. S., Brady, A., Bunn, C., Donnan, P.T., Fenwick, E., Grieve, E., Leishman, J., & Miller, E. (2014). A gender-sensitised weight loss and healthy living programme for overweight and obese men delivered by Scottish Premier League football clubs (FFIT): a pragmatic randomised controlled trial. The Lancet, 383(9924), 1211-1221.

Hunt, K., Wyke, S., Bunn, C., Donnachie, C., Reid, N., & Gray, C. M. (2020). Scale-up and scale-out of a gender-sensitized weight management and healthy living program delivered to overweight men via professional sports clubs: the wider implementation of Football Fans in Training (FFIT). International Journal of Environmental Research and Public Health, 17(2), 584.

Norman, R., Cronin, P., & Viney, R. (2013). A Pilot Discrete Choice Experiment to Explore Preferences for EQ-5D-5L Health States. Applied Health Economics and Health Policy, 11(3), 287-298. doi:10.1007/s40258-013-0035-z

Quested E, Kwasnicka D, Thøgersen-Ntoumani C, Gucciardi DF, Kerr DA, Hunt K, et al. Protocol for a gender-sensitised weight loss and healthy living programme for overweight and obese men delivered in Australian football league settings (Aussie-FIT): A feasibility and pilot randomised controlled trial. BMJ Open. 2018;8(10):e022663.

Steeves, J. A., Bowles, H. R., Mcclain, J. J., Dodd, K. W., Brychta, R. J., Wang, J., & Chen, K. Y. (2015). Ability of thigh-worn ActiGraph and activPAL monitors to classify posture and motion. Medicine and science in sports and exercise, 47(5), 952.

White, I. R., Horton, N. J., Carpenter, J., & Pocock, S. J. (2011). Strategy for intention to treat analysis in randomised trials with missing outcome data. *BMJ*, 342, d40. https://doi.org/10.1136/bmj.d40
